# Chemotherapy-Treated Breast Cancer Cells Activate the WNT Signaling Pathway to Enter a Diapause-Like Early Persister State

**DOI:** 10.1158/0008-5472.CAN-24-4165

**Published:** 2025-10-21

**Authors:** Youssef El Laithy, Willy Antoni Abreu De Oliveira, Anirudh Pabba, Alessandra Qualizza, Gwenny Cosemans, Paula Garcia-Diaz, François Richard, Paraskevi Athanasouli, Carla Rios Luci, Wout De Wispelaere, Larissa Mourao, Siân Hamer, Stijn Moens, Anchel De Jaime-Soguero, Maria Francesca Baietti, Stefan J. Hutten, Jos Jonkers, Stephen-John Sammut, Stefaan Soenen, Colinda L.G.J. Scheele, Alejandra Bruna, Christine Desmedt, Daniela Annibali, Frederic Lluis

**Affiliations:** 1 Department of Development and Regeneration, Stem Cell Institute, KU Leuven, Leuven, Belgium.; 2 Department of Oncology, Laboratory for Translational Breast Cancer Research, KU Leuven, Leuven, Belgium.; 3 Department of Imaging and Pathology, NanoHealth and Optical Imaging Group, KU Leuven, Leuven, Belgium.; 4 Gynecological Oncology, Department of Oncology, KU Leuven, Leuven, Belgium.; 5 Laboratory of Intravital Microscopy and Dynamics of Tumor Progression, Department of Oncology, VIB-KU Leuven Center for Cancer Biology, Leuven, Belgium.; 6 Preclinical Modelling of Pediatric Cancer Evolution, Institute of Cancer Research (ICR), London, United Kingdom.; 7 TRACE – Laboratory for RNA Cancer Biology, Department of Oncology, KU Leuven, Leuven, Belgium.; 8 Division of Molecular Pathology, The Netherlands Cancer Institute, Amsterdam, the Netherlands.; 9 Oncode Institute, Amsterdam, the Netherlands.; 10 Breast Cancer Now Toby Robins Research Centre, Institute of Cancer Research (ICR), London, United Kingdom.; 11 The Royal Marsden Hospital, NHS Foundation Trust, London, United Kingdom.

## Abstract

Cancer cells can acquire a reversible, dormant drug-tolerant persister state mimicking embryonic diapause to evade therapy pressure. Deciphering the precise mechanisms driving cancer cells into or out of a diapause-like persister cell state could provide strategies to overcome resistance. In this study, we showed that following chemotherapy, diverse therapeutic agents converge on WNT pathway activation to induce a *de novo* diapause-like cell state across various triple-negative breast cancer cell line, xenograft, and patient-derived organoid models. Among early persister cells, only transcriptionally WNT-active persisters exhibited the transcriptional and functional characteristics typical of diapause-like cells, including a negative correlation with MYC transcriptional activity and reversible restricted proliferation. The WNT signaling pathway functioned as both an inducer and biomarker of the diapause-like early persister cell state in both parental (chemotherapy-naïve) and chemotherapy-treated cells. Entry into and exit from the diapause-like early persister cell state was triggered by the transcriptional upregulation of components essential for canonical WNT ligand secretion. A combinatorial treatment strategy inhibiting WNT ligand secretion alongside chemotherapy effectively targeted the early mechanisms underlying the acquisition and enrichment of a diapause-like cell phenotype. These findings reveal WNT pathway activation as an early event that leads to a reversible diapause-like persister state and highlight the potential of targeting this axis to prevent the development of drug-resistant populations before they are firmly established.

**Significance:**

WNT signaling is a crucial driver and biomarker of a reversible, dormant, diapause-like persister state in breast cancer cells, offering insights that could transform therapeutic strategies to disrupt tumor persistence.

## Graphical Abstract

**Figure:**
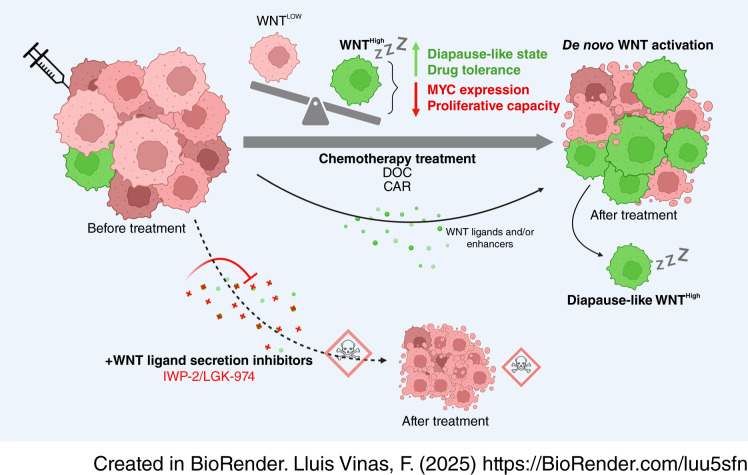

## Introduction

Drug tolerance in cancer cells often leads to treatment failure and disease relapse. Nongenetic mechanisms, including transcriptional rewiring, altered metabolic states, and suppression of apoptosis, contribute to the development of drug-tolerant states. Accumulating evidence indicates that a subset of cancer cells can evade chemotherapy-induced cell death by entering a reversible, slow-proliferating or dormant state resembling embryonic diapause (1, 2). Although this diapause-like state primarily arises under therapeutic pressure, it has also been observed as a small subpopulation within the parental tumor (3–5). Cancer diapause-like cells are often referred to as persister or drug-tolerant persister (DTP) cells, as their slow-proliferating phenotype enables them to persist through chemotherapy (1, 2). However, it is unclear whether all persister cells adopt a true diapause-like state. Identifying diapause-like cells among other drug-tolerant states within both parental and chemotherapy-treated populations remains challenging. Furthermore, the molecular mechanisms and signaling pathways that specifically regulate entry into and exit from a diapause-like persister state are largely unknown.

In mouse embryonic stem cells, dormancy or slow proliferation is influenced by mTORC1/2 inhibition (6). Alternatively, genetic depletion of *c-Myc* and *n-Myc* in mouse embryonic stem cells induces a pluripotent dormant state that mimics diapause (7, 8). Similarly, in cancer cells, pharmacologic inhibition or depletion of MYC promotes a diapause-like state marked by reduced proliferation and increased therapy resistance. Consistent with this, cancer diapause-like persister cells exhibit a negative correlation with transcriptional MYC hallmark expression, underscoring MYC’s central and pivotal role in this process (2).

Cancer DTP cells exhibit features of epithelial–mesenchymal transition (EMT), which is associated with poor drug responsiveness, a senescence-like gene signature, and enhanced stemness (9–11). Furthermore, upon discontinuation of treatment—commonly referred to as a drug holiday—diapause-like persister cells resume growth and proliferation, and their progeny retain sensitivity to chemotherapy (12). Only a few biomarkers have been associated with persister cells enriched after chemotherapy, including growth and differentiation factor 15 as well as developmental and pluripotency-associated factors such as aldehyde dehydrogenase, CD133, CD44, and KDM5B (13). Whereas these biomarkers may help identify stress tolerance as a feature of broad drug-tolerant states, specific biomarkers for the diapause-like persister state are still lacking.

Many patients with triple-negative breast cancer (TNBC) initially benefit from preoperative (neoadjuvant) chemotherapy (NAC); however, about 30% to 50% develop resistance, leading to poor overall survival rates (14, 15). Drug resistance has conventionally been attributed to the selection of preexisting resistant (stem) cell populations (intrinsic or Darwinian selection; ref. 16). However, recent research using genomic and transcriptomic deep sequencing of matched longitudinal (before and after NAC treatment) TNBC patient and patient-derived xenograft samples has also highlighted the role of acquired (drug-induced) resistance during chemotherapy (17, 18). Interestingly, residual TNBC tumors treated with NAC do not exhibit an enrichment of a breast cancer stem cell (BCSC) population (CD24^Low^/CD44^High^ cells; ref. 18). DTP cell enrichment has been demonstrated across distinct chemotherapeutic and targeted agents (3–5, 19); however, it remains unknown whether the emergence of a persister cell state converges on common downstream molecular mechanisms, even when induced by distinct chemotherapeutic agents with divergent proapoptotic mechanisms of action.

In this study, we aimed to delineate the early molecular mechanisms driving the emergence and/or enrichment of cancer persister cells in response to therapeutic pressure. Our findings demonstrate that treatment pressure induces *de novo* WNT transcriptional activation, regardless of the chemotherapeutic agent used [docetaxel (DOC) or carboplatin (CAR)]. This activation of the WNT signaling pathway by cytotoxic treatment was not limited to *in vitro* 2D-cultured TNBC cell lines but was also consistently observed in 3D-cultured TNBC patient-derived organoid (PDO) models and *in vivo* xenograft models. Among all early persister cells, we show that only WNT-active (WNT^High^), in contrast to WNT-inactive (WNT^Low^) persister cells, acquire true transcriptional and functional features characteristic of diapause, such as reversible reduced proliferation and negative MYC transcriptional activity. Notably, WNT pathway activation in parental naïve cells replicates the diapause-like cellular state even in the absence of chemotherapeutic intervention. Thus, we conclude that the transcriptional activation of the WNT signaling pathway not only initiates but also acts as a distinct biomarker for the early development of the diapause-like cell phenotype both in parental and chemotherapy-treated samples.

We find that diapause-like persister cell enrichment during the early stages of chemotherapy is driven by increased expression of WNT ligands, R-spondins (RSPO), and molecules involved in WNT ligand secretion. We show that targeting the diapause-like cell population in parental samples by WNT ligand secretion inhibition does not mitigate *de novo* diapause-like early persister cell enrichment once chemotherapy pressure is applied. In contrast, concomitant inhibition of WNT ligand secretion alongside chemotherapy treatment significantly reduces diapause-like persister cell population and consequently sensitizes TNBC cell lines, xenograft models, and PDO models to chemotherapy.

## Materials and Methods

### Ethics declaration

All xenograft animal experiments performed were approved by the Ethics Committee at KU Leuven under the ethical approval codes P055/2022 and P016/2023.

PDO models used in this study were established from freshly resected tumor tissues obtained from patients with TNBC at the Antoni van Leeuwenhoek Hospital. The study was approved by the Institutional Review Board (NKI-B17PRE), and the subjects provided informed written consent.

All cell lines used in this study are approved for use by the Ethics Committee at the KU Leuven University Biobank under the code S65166.

### TNBC cell line culture

MDA-MB-231 (ATCC-HTB-26, RRID: CVCL_0062; female) and MDA-MB-468 (ATCC-HTB-132, RRID: CVCL_0419; female) were authenticated by the supplier and maintained in high-glucose DMEM (Gibco, 41965039) supplemented with 10% (v/v) FBS, 1 mmol/L sodium pyruvate (Gibco, 11140035), 100 μg/mL penicillin–streptomycin (Gibco, 15140163), and 0.01 mmol/L 2-mercapthoethanol (Gibco, 31350010).

The PDC-BRC-101 cell line (patient-derived xenograft–derived cell line; female) was obtained from collaborators, Daniela Anibali and Stijn Moens (Amant Laboratory – Gynecological Oncology) – KU Leuven, and maintained in OCMI media (20) composed of a 1:1 mixture of Medium 199 (Gibco, 31150022) and DMEM F-12 (Gibco, 11320074) supplemented with 10% (v/v) FBS, 100 μg/mL penicillin–streptomycin, 20 μg/mL insulin (Sigma/Merck, I9278), 25 ng/mL cholera toxin subunit B (Sigma/Merck, C9903-.5MG), 0.5 μg/mL hydrocortisone (Sigma/Merck, H0888-1G), and 10 ng/mL EGF (Stem Cell Technologies, 78006.1).

All cell lines were cultured in 84 × 20 mm (D × H) tissue culture–treated dishes at 37°C and 5% CO_2_ and maintained at 70% to 80% confluency. For cell line passaging and plating, 1× PBS (Gibco, 10010-015) was used as a washing solution followed by dissociation using 0.25% trypsin-EDTA (Gibco, 25200-056) and cell pelleting by centrifugation for 4 minutes at 300 × *g* (0.3RCF). Cells were counted manually via the BRAND counting chamber Neubauer improved (Sigma-Aldrich/Merck, BR717810-1EA) under a 10× objective lens using a Leica DMi inverted microscope. The same microscope, equipped with a 2.5 Megapixel HD Microscope Camera Leica MC120 HC, was used to obtain images of cultured cancer cell lines. Unless specified otherwise, cancer cell lines were plated according to the following seeding densities: $7.3\;x\;10^{3}\;\frac{cells}{cm^{2}}$ and $10.5\;x\;10^{3}\;\frac{cells}{cm^{2}}$ (MDA-MB-231 and MDA-MB-468/PDC-BRC-101, respectively). Cell lines were stored at –180°C at low passage (<passage 5) and, for experiments, were used after 2 to 3 passages following thawing, corresponding to approximately 7 to 10 days in culture.

Cell lines were routinely checked (once a month) for *Mycoplasma* contamination using the innuPREP DNA Mini Kit 2.0 (Westburg, 845-KS-1042010) according to the manufacturer’s instructions (eukaryotic cell culture protocol).

### Chemotherapeutic and small molecule treatment of TNBC cell lines and PDO models

Cell lines were treated with increasing concentrations of DOC (Taxotere, 0–144 nmol/L) and CAR (Carbosin, 0–1,600 μmol/L) for 72 hours. Cell metabolic activity, (reflecting cell number and viability) was assessed using the (thiazolyl blue tetrazolium bromide assay (Sigma/Merck, M5655-500mg) according to the manufacturer’s instructions, and sigmoidal dose–response curves were generated to calculate the mean IC_50_ values of each drug that were used in the subsequent study. Chemotherapeutic agents were obtained from the pharmacy of Universitair Ziekenhuis Leuven.

For WNT pathway stimulation, CHIR99021 (CHIR – Sigma/Merck, SML1046) and 6-bromoindirubin-3′-oxime (BIO – Sigma/Merck, B1686-5MG) were used at 8 and 3 μmol/L, for CHIR and BIO, respectively.

### Lentiviral particle production and transduction

Lentiviruses were produced according to the RNAi Consortium (TRC) protocol available from the Broad Institute (https://portals.broadinstitute.org/gpp/public/resources/protocols). In brief, $7\;x\;10^{5\;}$ HEK293T (RRID: CVCL_0063) cells were seeded per well in six-well plates and transfected the following day with 750 ng pCMV-dR8.91 (RRID: Addgene_202687), 250 ng pCMV-VSV-G (RRID: Addgene_8454), and 1 μg of the specific lentiviral plasmid/construct using FugeneHD (Promega, E2311) in Opti-MEM (Gibco, 31985070). One day after, the culture medium was refreshed. The same day, lentivirus-recipient cells were plated in six-well plates at their respective concentrations (see cell line culture). Lentivirus-containing medium was collected from HEK293T cells 48 and 72 hours after transfection and added to recipient cancer cells after filtration using a 0.45-μm filter (VWR-Corning, 431220). Forty-eight hours after infection, recipient cancer cells were washed thoroughly with PBS, medium refreshed, and the appropriate selection antibiotics applied until the selection process was completed.

WNT transcriptional reporters TOPGFP (7xTcf-eGFP // SV40-PuroR), TOPFLASH (7xTcf-FFluc), and mCherry-TOPGFP (7xTcf-eGFP//SV40-mCherry) were obtained from Addgene (#24305 – RRID: Addgene_24305, #24308 – RRID: Addgene_24308, and #24304 – RRID: Addgene_24304, respectively). WNT transcriptional reporter dTOPGFP (dTGP) was gifted to us from the Moon Lab, University of Washington.

For Porcupine (PORCN) short hairpin RNA (shRNA)-mediated silencing, we used the MISSION Lentiviral shRNA (Sigma-Aldrich/Merck, SHCLNG – clones, TCRN000153848 and TCRN000157366) and the MISSION pLKO.1-puro Non-Target shRNA Control Plasmid DNA (SHC016-1EA) as a negative control in experiments.

### qRT-PCR and gene expression analysis

For qRT-PCR, total RNA was extracted (from TNBC cell lines or cryopreserved tumor tissue) using the GenElute Mammalian Total RNA Miniprep Kit (Sigma/Merck, RTN350-1KT) according to the manufacturer’s instructions with an additional step of DNA digestion using the On-Column DNase I Digestion Set according to the manufacturer’s instructions (Sigma/Merck, DNASE70). cDNA was synthesized from 500 ng of total RNA using the BIORAD iScript cDNA Synthesis kit (Bio-Rad, cat. #1708891) according to the manufacturer’s instructions. qRT-PCR reactions were set up in technical triplicates with Platinum SYBR Green qPCR SuperMix-UDG (Invitrogen, 11733-046) on a ViiA7 Real-Time PCR System (Thermo Fisher Scientific). Expression levels were normalized to two housekeeping genes, *RPL19* and *GAPDH*, to determine ΔCT values. Statistical testing of differences in expression levels between samples was carried out based on relative expression values ($2^{-\Delta CT}$). In some figures, gene expression values are represented as fold change (FC) for convenience of interpretation, although statistical testing was performed on relative expression values ($2^{-\Delta CT}$).

### SDS-PAGE and Western blot analysis

TNBC cell lines were washed with PBS and collected/pelleted by centrifugation. Whole-cell lysates were obtained via mechanical lysis using a needle (VWR-TERUMO, AN2138R1) and RIPA cell lysis buffer (Sigma/Merck, R0278-50mL) supplemented with a cocktail of 1:100 phosphatase inhibitor cocktails 2 and 3 (Sigma/Merck, P5726-1ML and P0044-1ML, respectively) and 1:100 protease inhibitor cocktail (Sigma/Merck, 11873580001). Samples were placed on a rotation wheel for a minimum of 30 minutes at 4°C, after which, they were centrifuged at 16,000 × *g* for 10 minutes at 4°C. The supernatant from the lysates was collected, and protein concentration was determined using the Bradford Assay (Bio-Rad, 5000006). For SDS-PAGE, 20 μg of protein were mixed with 4× Laemmli buffer (240 mmol/L Tris/HCL, pH 6.8, 8% SDS, 0.04% bromophenol blue, 5% 2-mercaptoethanol, and 40% glycerol) and denatured for 5 minutes at 95°C prior to electrophoretic protein separation. Resolved protein extracts were transferred to polyvinylidene difluoride membranes (Bio-Rad, 162-0177). Transfer success was assessed with Ponceau S solution, and membranes were blocked with 5% nonfat milk or 5% BSA in TBS with 0.1% Tween-20 for 60 minutes. After blocking, membranes were incubated with primary antibodies at 4°C overnight. The day after, membranes were washed 3 times with TBS–Tween-20 for 10 minutes and incubated with secondary horseradish peroxidase–conjugated antibodies. Immunolabeled proteins were detected using SuperSignal West Pico Chemiluminescent Kit (Fisher Scientific, 34077) on autoradiography film (Santa Cruz, SC-201697). The primary antibodies used were active rabbit anti-nonphosphorylated β-catenin (Cell Signaling Technology, #19807S – RRID: AB_2650576), rabbit anti-PORCN (Novus Biologicals, NBP1-59677 – RRID: AB_11025374), and rabbit anti–WNT-2b (Abcam, ab178418). Mouse anti–β-actin (Santa Cruz Biotechnology, sc-47778 – RRID: AB_626632) was used as a loading control.

### Flow cytometry

For WNT activation assessment, cells were washed with PBS and collected/pelleted by centrifugation. Cells were resuspended in PBS + 2% FBS and counterstained with 5 μg of DAPI (1:1; final concentration 2.5 μg/mL; Sigma/Merck, D9542-10mg) to eliminate dead cells before running through the flow cytometer. Cell lines lacking any of the previously described WNT transcriptional reporters were used as gating controls.

For immunolabeling of CD44 and CD24, cells were detached, washed twice in PBS with 4% FBS, and incubated with CD44-PE (BD Pharmigen, 555479 – RRID: AB_395871) and CD24-APC (Thermo Fisher Scientific–Life Technologies, 17-0247-42 – RRID: AB_10718833) antibodies according to manufacturers’ specifications at room temperature. After incubations, cells were washed twice in PBS with FBS and resuspended in PBS containing 4% FBS and 100 nmol/L of DAPI. Cells incubated with PE- and APC-conjugated isotype antibodies, and single-stained cells were used as gating controls.

For Annexin V apoptosis analysis, cells were washed with PBS and collected/pelleted by centrifugation. Cells were resuspended in 1× Annexin V binding buffer (BD Pharmigen, 51-66121E) and incubated at room temperature in the dark for 15 minutes with APC-conjugated Annexin V (Thermo Fisher Scientific–eBioscience, BMS306APC-100). After incubation, cells were diluted in 1× binding buffer supplemented with 100 nmol/L of DAPI before running through the flow cytometer. Unstained and single-stained (Annexin V–only or DAPI-only stained) cells were used as gating controls.

To obtain chemotherapy-induced WNT^High^ and WNT^Low^ cells, cells were washed with PBS and collected/pelleted by centrifugation. Cells were resuspended in PBS + 4% FBS and counterstained with 5 μg of DAPI (1:1; final concentration 2.5 μg/mL) to eliminate dead cells before running through the SONY MA900 Multi-Application Cell Sorter. Depending on the application, $2-3\;x\;10^{5}$ cells were sorted (based on their GFP expression) into 1.5 mL Eppendorf tubes (with 300 μL of PBS + 4% FBS) and either used for RNA extraction and gene expression analysis or for reculturing.

For immunostaining of proliferation marker Ki-67, cells were washed with PBS and collected/pelleted by centrifugation. Cells were fixed with ice-cold 70% ethanol. Samples were washed with PBS + 2% FBS and blocked with 5% donkey serum (Jackson ImmunoResearch, 017-000-121 – RRID: AB_2337258) at room temperature for 30 to 60 minutes. Cells were repelleted by centrifugation, repeatedly washed with PBS + 2% FBS, and incubated with the Ki-67 recombinant rabbit mAb (SP6; Thermo Fisher Scientific, MA5-14520 – RRID: AB_10979488) at room temperature for 60 minutes. Cells were repelleted by centrifugation, washed repeatedly with PBS + 2% FBS, and incubated with a conjugated secondary antibody (donkey anti-rabbit – Alexa Fluor 647 – Thermo Fisher Scientific–Life Technologies, A31573 – RRID: AB_2536183) in the dark at room temperature for 30 minutes. Cells were repelleted by centrifugation, washed repeatedly with PBS + 2% FBS, and counterstained with 5 μg of DAPI (1:1; final concentration 2.5 μg/mL) before running through the flow cytometer. Unstained and single-stained (secondary antibody–only stained) cells were used as gating and analysis controls.

For immunostaining of active (nonphosphorylated) β-catenin, cells were washed with PBS and collected/pelleted by centrifugation. Cells were fixed with ice-cold 70% ethanol, after which, samples were fixed with PBS + 2% FBS and blocked with 5% donkey serum (in PBS) at room temperature for 30 to 60 minutes. Cells were repelleted by centrifugation, washed with PBS + 2% FBS, and incubated with active rabbit anti-nonphosphorylated β-catenin antibody (RRID: AB_2650576) at room temperature for 60 minutes. Cells were repelleted by centrifugation, washed with PBS + 2% FBS, and incubated with a conjugated secondary antibody (donkey anti-rabbit – RRID: AB_2536183) in the dark at room temperature for 30 minutes. Cells were repelleted by centrifugation, washed with PBS + 2% FBS, and counterstained with 5 μg of DAPI (1:1; final concentration 2.5 μg/mL) before running through the flow cytometer. Unstained and single-stained (secondary antibody–only stained) cells were used as gating and analysis controls.

Unless specified otherwise, all data were collected on a BD FACS Canto II at the KU Leuven Flow Cytometry Core and analyzed using FlowJo v10.6.2 (RRID: SCR_008520).

### Growth rate and doubling time analysis

For growth rate (μ) analysis, the following mathematical equation was used: $µ=\frac{\ln\left(\frac{N_{t}}{N_{0}}\right)}{\Delta t}\times 24h$, in which N_0_ is the number of cells seeded, N_t_ is the number of cells harvested/recorded, and Δt is the hours of growth.

For doubling time (t_d_) analysis, the following mathematical equation was used: $t_{d}=\frac{\ln\left(2\right)}{µ}\times 24h$.

### Conditioned media and coculture analysis

Conditioned media (CM) was collected from TNBC cell lines recovering from chemotherapy treatment (5 days of treatment and 1 week of recovery) and filtered using a 0.45-μmol/L filter to ensure removing cell debris. Filtered CM was concentrated 20× (20–1 mL) using Vivaspin centrifugal concentrator column with a molecular weight cutoff of 10 kDa (Sigma-Aldrich, Z614602-12EA). Filtered media were centrifuged for 45 minutes at 4°C. Concentrated CM was added to chemotherapy-naïve TNBC cell lines for 48 hours in a 1:1 dilution (concentrated CM:basal culture cancer media), and WNT activation levels were evaluated using FACS.

For coculture experiments, the MDA-MB-231 cell line was treated with either chemotherapeutic agent for 72 hours, after which, treatment was stopped and an equal number of chemotherapy-naïve MDA-MB-231-TGP.mC cells was plated in the same dish and cultured in basal culture cancer media. After 72 hours of coculture, WNT activation levels in the MDA-MB-231-TGP.mC cell line were evaluated using FACS.

### Immunofluorescence staining

TNBC cell lines were plated in μCLEAR 96-well plates (Greiner, 655090) at their respective seeded densities and treated with chemotherapy at their respective doses. Cells were washed with PBS and collected/pelleted by centrifugation and later fixed with 4% paraformaldehyde for 15 minutes at room temperature. Samples were permeabilized and blocked in a single step with 0.2% Triton-X and 5% donkey serum. Primary antibodies (active rabbit anti-nonphosphorylated β-catenin antibody – RRID: AB_2650576) were subsequently added and incubated overnight at 4°C. Next, cells were repeatedly washed with PBS and incubated with secondary antibodies (donkey anti-rabbit, Thermo Fisher Scientific, A31572 – RRID: AB_162543) for 40 minutes at room temperature in the dark. After 10 minutes of DAPI exposure (used for nuclei staining), cells were imaged using the high-throughput imager Operetta CLS system (PerkinElmer, HH16000000). Image analysis and quantification were performed using the Harmony High-Content Imaging and Analysis software v4.2 (PerkinElmer, HH17000001). Find nuclei – method B building block algorithm was used to segment nuclei based on DAPI signal to accurately assess nuclear β-catenin signal. Unstained and single-stained (secondary antibody–only stained) cells were used as gating and analysis controls.

### Cell line–derived xenograft establishment and *in vivo* live imaging analysis

To establish cell line–derived xenograft models, $1\;x\;10^{6}$ MDA-MB-231 TOPFLASH cells were engrafted subcutaneously (1:1 PBS: growth factor–reduced Matrigel) into the right flank of female NMRI-Foxn1 mice (4–6 weeks old) to form a solid tumor. Upon observation of visible/palpable solid growth, tumor volumes were measured using digital calipers (and calculated as $L\;\times \;W\;\times \;\frac{\pi }{6}$, in which *L* is length and *W* is width). Animals were randomly assigned to one of three (or six) treatment groups (*n* = 7–8 mice per group), with an average tumor volume of 150 mm^3^ per group. DOC (15 mg/kg) and CAR (100 mg/kg) were administered via intraperitoneal injection once weekly (1 cycle) for a total of three cycles (3 weeks). LGK-974 (2 mg/kg) was administered daily via oral gavage for a total of three cycles (3 weeks). For assessment of WNT activation dynamics, animals were subjected to live bioluminescent imaging before and 24, 48, and 72 hours after chemotherapeutic administration. For live bioluminescent imaging, animals were injected intraperitoneally with the luciferase substrate D-luciferin (200 μL of 15 mg/mL, assuming an average animal weight of 24–26 g; PerkinElmer, 122799) and incubated for 10 minutes at room temperature before images were taken using IVIS Spectrum In Vivo Imaging System (PerkinElmer). WNT activation signal was calculated as the bioluminescent signal captured by the IVIS Spectrum normalized to the tumor volume recorded per animal. Analysis of bioluminescent images was performed via the Aura software v4.0.0. Tumor volume was recorded every 48 hours, and body weight was closely monitored throughout the treatment course and recorded every 72 to 96 hours using an automatic scale. All animals were euthanized at the end of the treatment course, and tumors (when available) were resected/collected for downstream analyses.

Group size was selected based on a power input (0.8), six treatment groups [including vehivle (VEH)/control groups], and a minimum of seven measurements. The software G power (v3.1.9.2) was used to calculate and determine the sample/group size.

### RECIST analysis

RECIST analysis was performed using tumor volumes measured and recorded (as described previously) at the onset of treatment and at the end of treatment (day of sacrifice). Relative tumor volume (RTV) was calculated by dividing the recorded volume at the end of treatment by the recorded volume at the onset of treatment. Response to therapy was based on the RECIST-based criterion: complete response (CR), partial response (PR), stable disease (SD), and progressive disease (PD); CR: RTV = 0, PR: 0 < RTV ≤ 0.657, SD: 0.657 < RTV ≤ 1.728, and PD: RTV > 1.728.

### Next-generation mRNA sequencing

Total RNA was obtained from cells using the GenElute Mammalian Total RNA Miniprep Kit (Sigma, RTN350-1KT). RNA sequencing (RNA-seq) libraries were prepared using 750 ng of total RNA using the KAPA Stranded mRNA-Seq Kit (Roche, 8098123702) according to the manufacturer’s specifications. A total of 100 nmol/L of KAPA single-index adapters (Roche, KK8702) were added to the A-tailed cDNA, and the libraries underwent 10 cycles of amplification. Agencourt AMPure XP beads (Beckman Coulter, A63880) were used for the 1X library clean-up. The fragment size of the libraries was assessed using the Agilent Bioanalyzer 2100 with the High Sensitivity DNA Kit (Agilent, 5067-4626). The concentration of the libraries was measured using the High Sensitivity QuBit Kit (Invitrogen, Q33230). Each library was diluted to 4 nmol/L and pooled for single-end, 50-bp sequencing on an Illumina HiSeq 4000 with 20 to 27 million reads per sample (22 million reads on average).

### Bulk mRNA sequencing analysis

FASTQ files generated from the sequencing (sequencing run) were sent for downstream processing. Adapters were trimmed using Trimmomatic v0.39, and the trimmed FASTQ file was aligned to the GRCh38 genome (hg38) using the STAR aligner v2.7.10. Gene counts, gene annotation, and sample read characteristics were obtained by applying standard filters within featureCounts from the subread package v2.0.3. Gene counts were then normalized using variance-stabilizing transformation. *Z*-scores used to describe the gene expression distribution across samples were calculated using median absolute deviation, whereas the heatmaps comparing *z*-scores between samples were created using pheatmap v1.0.12. Differential gene expression analysis was performed using DESeq2 (21), and batch effects were accounted for in the model comparing the WNT^High^ versus WNT^Low^ cohort. Volcano plots were created using EnhancedVolcano v1.18.0 using custom settings of |log_2_ FC|cutoff = 0.6 and pCutoff = 0.05. Gene set variation analysis (GSVA) was performed using GSVA v1.48.3. Signature scores for the caspase-3/apoptosis (22) and diapause-DTP signatures (Supplementary Table S5) were calculated after the gene counts were transformed using both log_2_(x) +1 and variance-stabilizing transformation methods. Box plots comparing the signature score(s) distribution between WNT^High^ and WNT^Low^ samples between treatment conditions were created using ggplot2 v3.4.3. Forest plots for regression analysis were created using forestplot v3.1.3. Analyses following the gene count extraction were all performed in R (23) v4.3.0.

### Gene set enrichment analysis of publicly available datasets

To identify sets of genes associated with a WNT-active (WNT^High^) signature, we performed differential gene expression on the trimmed mean of M-values (TMM) normalized gene counts obtained from the bulk mRNA sequencing (mRNA-seq) analysis using the edgeR R package (v4.2.2). We included treatment (DOC or CAR) as a covariate within the design matrix and selected genes that were differentially expressed at a log FC cutoff of 0.5. We tested the enrichment of this WNT signature, together with a diapause-DTP and MYC hallmark gene set in the GSE123845 Gene Expression Omnibus (GEO) dataset, which contains RNA-seq dataset acquired from longitudinally paired breast cancer during neoadjuvant treatment. We retained samples with expression data, which had a pre-therapy sample acquired, and one further sample acquired during treatment. Signature enrichment was performed on log-transformed transcripts per million (TPM) expression data using the single-sample singscore R package (v1.24.0), normalizing against 10 genes known to have a stable expression profile in pan-cancer datasets (*RBM45*, *NRF1*, *BRAP*, *WDR33*, *CNOT2*, *TIAL1*, *CIAO1*, *TARDBP*, *ZNF207*, and *HNRNPK*).

### PDO culture, treatment, and analysis

R1-IDC113 and R2-IDC159A PDO lines were gifted by our collaborator, Laboratory of Colinda Scheele – VIB-KU Leuven. Both PDO lines were maintained in growth factor–reduced type 2 Cultrex (Biotechne/R&D Systems, 3533-010-02) with phenol red–free DMEM/F-12, HEPES (Gibco, 11039021) supplemented with 10 mmol/L nicotinamide (Sigma/Merck, N0636-100G), 1.25 mmol/L N-acetyl-L-cystine (Sigma/Merck, A9165-5G), 500 ng/mL hydrocortisone, 100 nmol/L β-estradiol (E8875-250MG), 500 nmol/L SB202190 (Stem Cell Technologies, 72632), 500 nmol/L A83-01 (Stem Cell Technologies, 72022), 5 μmol/L Y-27632 (Stem Cell Technologies, 72304), 50 μg/mL Primocin (Invivogen, ant-pm-05), 10 μmol/L forskolin (Sigma/Merck, F3917-10MG), 1X B27 (50X – Thermo Fisher Scientific, 17504044), 100 ng/mL r-Noggin (Stem Cell Technologies, 78060), 5 ng/mL FGF-10 (Stem Cell Technologies, 78037), 37.5 ng/mL heregulin B-1 (Peprotech, 100-03), 5 ng/mL EGF, 5 ng/mL FGF-7 (Peprotech, 100-19), and 100 μg/mL penicillin–streptomycin.

Both PDO lines were cultured in-24 well cell culture microplates – 22 mm × 20 mm (D x H) – at 37°C and 5% CO_2_ and maintained at 70% to 80% confluency. For passaging and plating, (ice-cold) 1× PBS (Gibco, 10010-015) was used to wash and dissociate the basement membrane extract, followed by single-cell enzymatic dissociation using 0.05% trypsin-EDTA (Gibco, 25200-056) and cell pelleting by centrifugation for 5 minutes at 1,500 rpm (4°C). Cells were counted manually via the BRAND counting chamber Neubauer improved (Sigma-Aldrich/Merck, BR717810-1EA) under a 10× objective lens using a Leica DMi inverted microscope. The same microscope, equipped with a 2.5 Megapixel HD Microscope Camera Leica MC120 HC, was used to obtain images of cultured PDO lines. Unless specified otherwise, both PDO models were plated according to the following seeding density: $5.9\;x\;10^{2}\;\frac{cells}{mm^{2}}$. PDO lines were stored at –180°C at low passage (<passage 4) and, for experiments, were used after 2 to 3 passages following thawing, corresponding to approximately 10 to 15 days in culture.

To determine working chemotherapy drug concentrations, PDO lines were treated with increasing concentrations of DOC (Taxotere, 0–512 nmol/L) and CAR (Carbosin, 0–6,400 μmol/L) for 96 hours. Cell metabolic activity, reflecting cell number and viability, was assessed using the CellTiter-Glo 3D Cell Viability Assay (Promega, G9682), and sigmoidal dose–response curves were generated to calculate the mean IC_50_ values of each drug that were used in the subsequent study. Chemotherapeutic agents were obtained from the pharmacy of Universitair Ziekenhuis Leuven.

PDO lines were routinely checked (once a month) for *Mycoplasma* contamination using the innuPREP DNA Mini Kit 2.0 (Westburg, 845-KS-1042010) according to the manufacturer’s instructions (eukaryotic cell culture protocol).

### Statistical analysis

All data were analyzed using GraphPad Prism (v8.0.1), except for mRNA-seq–derived data and transcriptomic datasets. Unless otherwise specified, comparisons between two groups were tested for statistical significance using unpaired *t* tests. Comparisons between multiple groups were performed using a one-way ANOVA. Comparisons between multiple groups across multiple time points were performed using two-way ANOVA. Unless specified otherwise, all statistical testing was corrected for multiple comparisons using the Holm–Sidak method when comparing samples based on experimental design. Unless otherwise specified, *n* refers to independent biological experiments. For the reader’s convenience, all statistical tests, sample sizes, and corrections are indicated in the figure legends.

For mRNA-seq–derived data, regression analysis was performed to observe associations between outcomes (in-house gene signature scores/GSVA signature scores) and independent covariate (WNT^High^ vs. WNT^Low^) per treatment condition [CAR or DOC or untreated (UNT)] using lqmm v1.5.8 and quantreg v5.97 while accounting for batch effects.

## Results

### Distinct chemotherapy treatments converge on robust WNT/β-catenin pathway activation during early persister cell enrichment

To investigate common mechanisms driving early persister cell emergence across therapies, we modeled early persister formation *in vitro*. Three TNBC cell lines (MDA-MB-231, MDA-MB-468, and PDC-BRC-101) were treated with two chemotherapeutics: DOC, which stabilizes microtubules, and CAR, which induces DNA damage (24, 25). IC_50_ values at 72 hours were determined for each drug–cell line combination and used in subsequent experiments (Supplementary Table S1).

We next performed bulk transcriptomic analysis on viable/drug-tolerant (DAPI^−^) MDA-MB-231 cells treated with either DOC or CAR (Supplementary Fig. S1A). Gene set enrichment analysis (26) using MSigDB (27) datasets on differentially expressed genes (Supplementary Fig. S1B and S1C; Supplementary Table S2) between DOC or CAR versus UNT (FC > 1.5, *P* value ≤ 0.05) identified enrichment of hallmarks associated with stress response (apoptosis, p53 pathway, and IFNγ response; Supplementary Fig. S1D; Supplementary Table S3). Conversely, hallmarks associated with cell-cycle regulation, such as G2M checkpoint, DNA repair, MYC targets, and E2F targets were significantly downregulated under DOC and CAR treatment (Supplementary Fig. S1D; Supplementary Table S3). Interestingly, gene signatures and processes such as EMT and hypoxia, linked to tumorigenesis, chemoresistance, and the persister cell phenotype, were enriched in response to chemotherapeutic exposure (Supplementary Fig. S1E; Supplementary Table S3; refs. 28, 29).

**Figure 1.**
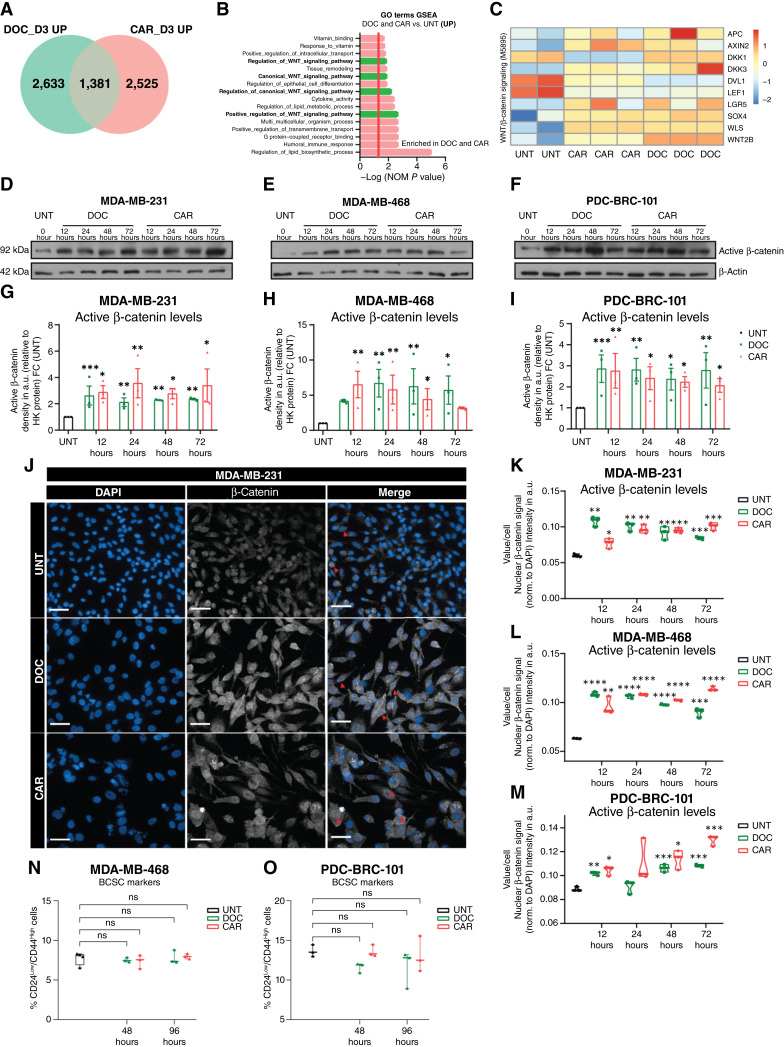
Distinct chemotherapy treatments converge on robust WNT/β-catenin pathway activation during early persister cell enrichment. **A,** Venn diagram of commonly upregulated (UP; 1,381) genes between DOC (2,633) and CAR (2,525) vs. UNT. **B,** Gene ontology (GO) processes from one-tailed gene set enrichment analysis (GSEA) of shared upregulated genes (**A**), ranked by positive normalized enrichment score. Red line indicates significance [−log(NOM *P* value) = 1.3]; highlighted terms relate to WNT/β-catenin signaling regulation. **C,** Heatmap of 10 WNT/β-catenin signaling genes in the MDA-MB-231 cell line treated with DOC or CAR for 72 hours, presented as Z-scores (selected from WNT/β-catenin signaling hallmark, GSEA). Data for **A–C** were obtained from bulk mRNA-seq of the MDA-MB-231 cell line treated with chemotherapy for 72 hours. **D–F,** Western blots of active (nonphosphorylated) β-catenin in MDA-MB-231 (**D**), MDA-MB-468 (**E**), and PDC-BRC-101 (**F**) cells treated with chemotherapy for 12 to 72 hours. **G–I,** Quantification of blots in **D–F**, displayed as FC to UNT; signals normalized to β-actin. Mean ± SEM. Multiple *t* tests, *n* = 3. **J,** Immunofluorescence of active β-catenin in MDA-MB-231 cells treated with chemotherapy for 72 hours. Scale bar, 50 μm. Images show zoomed-in regions of interest. **K–M,** Quantification of nuclear active β-catenin (normalized to DAPI, find nuclei – method M) in TNBC cell lines treated with chemotherapy for 12 to 72 hours. Violin plots, all points shown. Multiple *t* tests, Holm–Sidak correction, *n* = 3. **N** and **O,** Flow cytometry of %CD24^Low^/CD44^High^ cells in MDA-MB-468 (**N**) and PDC-BRC-101 (**O**) cell lines treated with chemotherapy for 48 or 96 hours. Box-and-whisker plots. Multiple *t* tests, Holm–Sidak correction, *n* = 3. *, *P* < 0.05; **, *P* < 0.01; ***, *P* < 0.001; ****, *P* < 0.0001; ns, not significant.

Gene ontology analysis of common enriched transcriptomic alterations among both chemotherapeutic agents (vs. UNT; Fig. 1A; Supplementary Tables S2 and S3) highlighted positive regulation of canonical WNT signaling, corroborated by enrichment in the expression of WNT target genes (*AXIN2* and *LGR5*) and upstream activators (*WLS* and *WNT2B*) of the pathway (Fig. 1B and C). Conversely, gene ontology analysis using commonly downregulated genes highlighted enrichment in processes related to cell-cycle regulation and progression (Supplementary Fig. S1F and S1G).

At the protein level, Western blotting confirmed elevated active (nonphosphorylated) β-catenin from 12 to 72 hours after treatment in all TNBC lines (Fig. 1D–I), with immunofluorescence showing increased nuclear localization (Fig. 1J–M; Supplementary Figs. S1H, S2A, and S2B). Expression of WNT target genes, *AXIN2* and *LGR5*, was also significantly upregulated after chemotherapy (Supplementary Fig. S2C–S2E).

Previous studies have highlighted the role of WNT signaling in maintaining BCSCs as a mechanism underlying WNT-mediated drug tolerance (25, 30–32). Interestingly, although we detected a small population of BCSCs (CD24^Low^/CD44^High^ cells) in parental cells, we observed no significant enrichment at either 48 or 96 hours of treatment in MDA-MB-468 and PDC-BRC-101 TNBC cell lines (Fig. 1N and O; Supplementary Fig. S2F and S2G). This aligns with previous findings indicating that chemotherapy does not enrich for BCSCs in TNBC (18).

Our data show that canonical WNT signaling is consistently upregulated across TNBC cell lines following both cytotoxic treatments. Importantly, this activation during early persister enrichment did not coincide with an expansion of the BCSC population, indicating that chemotherapy-induced WNT activity drives persister cell enrichment through alternative mechanisms.

### Parental and early chemotherapy-treated WNT^High^ persister cells display diapause-like cell properties

**Figure 2.**
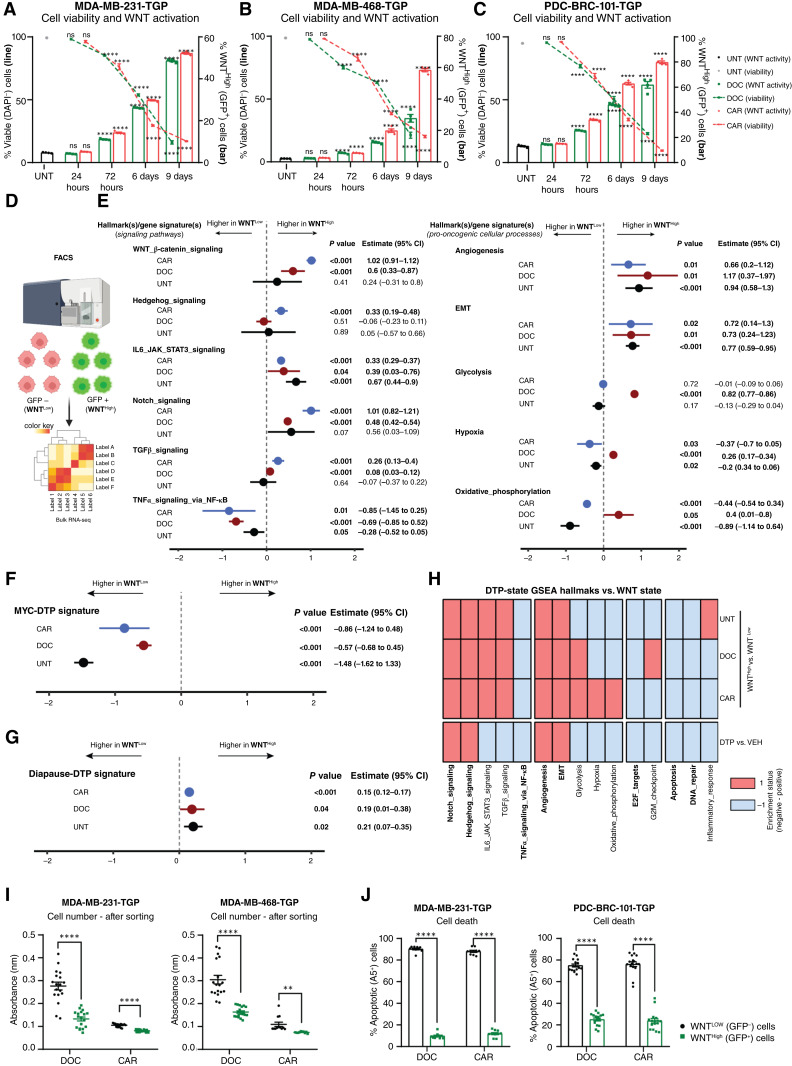
Parental and early chemotherapy-treated WNT^High^ persister cells display diapause-like cell properties. **A–C,** Flow cytometry of %WNT^High^ (GFP^+^) cells (bars, right *y*-axis) and %viable (DAPI^−^) cells (dashed line, left *y*-axis) in TNBC-TGP cell lines treated with DOC or CAR for 24 hours, 72 hours, 6 days, or 9 days. One-way ANOVA, Holm–Sidak correction, *n* = 4. **D,** Experimental setup for transcriptional comparison of sorted WNT^High^ vs. WNT^Low^ MDA-MB-231-dTGP cells treated with chemotherapy for 72 hours (bulk mRNA-seq). **E,** Forest plots showing association between gene signatures and WNT status in sorted WNT^High^ vs. WNT^Low^ cells from DOC, CAR, and UNT samples. Gene signatures include in-house signatures (22) and hallmark gene sets (MSigDB), analyzed using GSVA. Quartile regression used to observe the median change in rescaled gene signatures after accounting for batch effects. Signatures having a nonzero positive estimate indicate increased activity in WNT^High^ cells. **F** and **G,** Forest plots for MYC hallmark (upregulated) and diapause-DTP (1; downregulated) signatures vs. WNT status, analyzed as in **E**. **H,** Binary pattern analysis between transcriptional DTP cells (2) and WNT status of sorted WNT^High^/WNT^Low^ cells obtained from samples treated with CAR or DOC and UNT samples. Enrichment status score of −1 (light blue) indicates that a given hallmark/process is downregulated in DTPs or sorted WNT^High^ cells, whereas a score of 1 (red) indicates that a given hallmark/process is upregulated in DTPs or sorted WNT^High^ cells. Data for **E–H** were obtained from bulk mRNA-seq of sorted WNT^High^/WNT^Low^ of MDA-MB-TGP cells treated with DOC or CAR for 72 hours. **I,** Absorbance values of cellular metabolic activity indicating cell number in sorted MDA-MB-231-TGP and MDA-MB-468-TGP cell lines 1 week after sorting (initial treatment was 72 hours of chemotherapy). Multiple *t* tests, Holm–Sidak correction, *n* = 3. **J,** Flow cytometry of apoptotic (% Annexin V^+^) cells and corresponding WNT status (%WNT^High^ cells) in MDA-MB-231-TGP and PDC-BRC-101-TGP cell lines treated with chemotherapy for after 96 hours. Multiple *t* tests, Holm–Sidak correction, *n* = 5. Unless specified otherwise, all data are presented as the mean ± SEM. *, *P* < 0.05; **, *P* < 0.01; ***, *P* < 0.001; ****, *P* < 0.0001; ns, not significant. CI, confidence interval. **D,** Created in BioRender. Lluis Vinas, F. (2025) https://BioRender.com/yen63o9.

To characterize chemotherapy-induced WNT-active cells, we treated three TNBC clonal lines carrying a stable WNT transcriptional reporter (TOP-GFP/TGP cell lines, Supplementary Fig. S3A; ref. 33) with DOC or CAR. Chemotherapy treatment significantly reduced cell number and viability at 72 hours (Fig. 2A–C; dashed lines and Supplementary Fig. S3B–S3D) while significantly increasing both the percentage and levels (intensity) of WNT^High^ (GFP^+^) cells among viable/early drug-tolerant (DAPI^−^) cells compared with UNT conditions (Fig. 2A–C; bars and Supplementary Fig. S3E and S3F). Prolonged exposure (6–9 days) further increased the levels of overall cell death and concurrently elevated the percentage of transcriptional WNT^High^ cells (Fig. 2A–C), highlighting that, among early persister cells, only a subpopulation becomes transcriptionally WNT-active.

To delineate transcriptional discrepancies among early persister cells, we performed bulk RNA-seq on sorted WNT^Low^ and WNT^High^ populations from chemotherapy-treated MDA-MB-231-dTGP cells (Fig. 2D; ref. 34). GSVA from differentially expressed genes (FC > 1.5, *P* value ≤ 0.05) revealed numerous hallmark signatures differentially regulated between chemotherapy-sorted WNT^High^ versus WNT^Low^ early persister cells (Supplementary Fig. S3G and S3H; Supplementary Tables S4–S6). As expected, WNT signaling was significantly and positively associated with chemotherapy-sorted WNT^High^ populations (Fig. 2E; Supplementary Fig. S3I and S3J). Whereas a few signatures exhibited drug-dependent associations, most hallmarks followed similar associative trends across sorted DOC- and CAR-treated populations (Fig. 2E; Supplementary Fig. S3I and S3J). Developmental pathways, including hedgehog, Notch, IL6/JAK/STAT3, and TGFβ signaling, along with hallmarks linked to tumor progression, stemness capacity, and metastasis (e.g., angiogenesis and EMT), displayed a significant positive association with WNT^High^ populations (Fig. 2E; Supplementary Fig. S3I and S3J). Conversely, the TNFα signaling pathway via the NF-κB pathway exhibited a significant negative association with WNT^High^ cells (Fig. 2E), aligning with previous findings suggesting that active β-catenin can attenuate transcriptional NF-κB activity in breast cancer (35).

Contrary to reports linking WNT activation to proliferation and elevated MYC (36, 37), our analysis showed that WNT^High^ cells negatively correlated with proliferative signatures, including G2M checkpoint and E2F targets (Supplementary Tables S5 and S6), suggesting a halted proliferative state in WNT^High^ persister cells. Recent studies (1, 2) have shown that DTP cells suppress MYC and display an embryonic diapause-like transcriptional profile. Consistent with this, chemotherapy-sorted WNT^High^ cells, but not WNT^Low^ cells, were negatively associated with the MYC hallmark and positively associated with the Rehman and colleagues diapause-like signature (Fig. 2F and G; Supplementary Fig. S3I and S3J), highlighting the similarities between the transcriptomes of diapause-like persister cells and chemotherapy-sorted WNT^High^ cells. Furthermore, coordinated regulation of additional hallmarks (Notch, EMT, and angiogenesis; upregulated and E2F targets, DNA repair, and apoptosis; downregulated) was coequally recorded in diapause-like cells and chemotherapy-sorted WNT^High^ cells (Fig. 2H).

We next assessed whether these transcriptional features observed in chemotherapy-sorted WNT^High^ cells preexisted in UNT (i.e., parental cells) conditions (Supplementary Fig. S3K). WNT^High^ cells obtained from UNT conditions showed a positive (yet statistically nonsignificant) association with the WNT/β-catenin hallmark signature (Fig. 2E), consistent with our previous data highlighting lower levels of WNT signaling intensity in UNT samples (Supplementary Fig. S3F). Interestingly, parental-sorted WNT^High^ cells exhibited overall positive associations with developmental pathways, EMT, and angiogenesis while exhibiting significant negative associations with cell-cycle hallmarks, following a similar trend as chemotherapy-sorted WNT^High^ early persister cells (Fig. 2E; Supplementary Fig. S3L; Supplementary Tables S4–S6). Moreover, parental-sorted WNT^High^ cells displayed a significant negative association with the MYC hallmark signature (Fig. 2F, UNT), a significant positive association with the Rehman and colleagues embryonic diapause-like gene signature (Fig. 2G, UNT), and other matching hallmarks and processes (Fig. 2H, UNT), highlighting transcriptional WNT activity as a functional marker for early diapause-like persister cells even in chemotherapy-naïve conditions. The significance of these correlations becomes more pronounced in chemotherapy-treated conditions, in which transcriptional WNT activation is strongly exacerbated.

Functional assays confirmed reduced proliferation in chemotherapy-sorted WNT^High^ versus WNT^Low^ populations (Fig. 2I). Costaining of GFP (WNT activity) with Annexin V showed that WNT^High^ cells had lower apoptosis, indicating that treatment primarily eliminates WNT^Low^ cells while enriching WNT^High^ persisters (Fig. 2J).

Together, these findings demonstrate that only WNT^High^ cells, both in parental drug-naïve and chemotherapy-treated conditions, exhibit a *bona fide* diapause-like transcriptional and functional phenotype. Thus, not all early persisters are diapause-like, and WNT transcriptional activity serves as a distinct biomarker of this state.

### WNT pathway activation triggers reduced proliferation, mimicking a diapause-like state in parental TNBC cells

**Figure 3.**
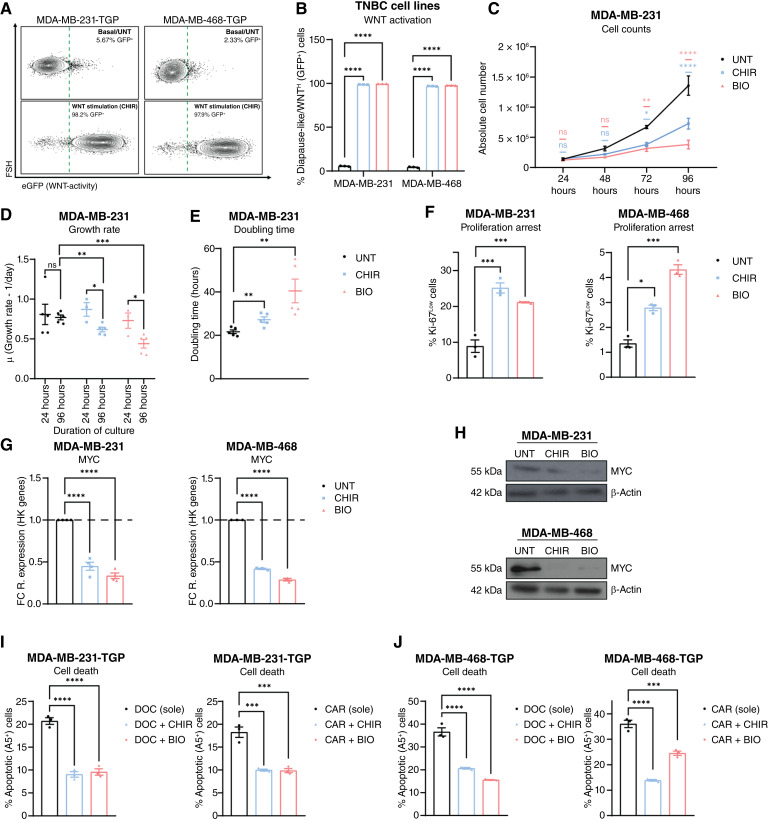
WNT pathway activation triggers reduced proliferation mimicking a diapause-like state in parental TNBC cells. **A,** Flow cytometry contour plots showing % diapause-like/WNT^High^ (GFP^+^) cells within viable (DAPI^−^) cells in MDA-MB-231-TGP and MDA-MB-468-TGP cell lines under basal (UNT; top) and WNT-stimulated (CHIR, 8 μmol/L, 72 hours; bottom) culture conditions. Right of dashed green line indicates diapause-like/WNT^High^ (GFP^+^) cells. **B,** Flow cytometry of % diapause-like/WNT^High^ (GFP^+^) cells in MDA-MB-231-TGP and MDA-MB-468-TGP cell lines under WNT-stimulatory conditions treated with CHIR (8 μmol/L) or BIO (3 μmol/L) for 72 hours. Two-way ANOVA and Tukey correction, *n* = 3. **C,** Absolute cell numbers of the MDA-MB-231 cell line treated with CHIR or BIO for 96 hours. Two-way ANOVA and Tukey correction, *n* = 3. **D,** Growth rate of the MDA-MB-231 cell line at 24 and 96 hours under UNT, CHIR, or BIO. Multiple *t* tests, Holm–Sidak correction, *n* = 3. **E,** Doubling time of MDA-MB-231 cell line at 96 hours under UNT, CHIR, or BIO. Multiple *t* tests, Holm–Sidak correction, *n* = 3. **F,** Flow cytometry of % cells in proliferation arrest (Ki-67^Low^ cells) in MDA-MB-231 and MDA-MB-468 cell lines after 96 hours of CHIR or BIO treatment. Multiple *t* tests, Holm–Sidak correction, *n* = 3. **G,** qRT-PCR of *MYC* in MDA-MB-231 and MDA-MB-468 cell lines after 72 hours of CHIR or BIO treatment, displayed as FC (to UNT) of 2^−dCt^ [relative to housekeeping (HK) genes]. Unpaired *t* tests on 2^−dCt^ values, *n* = 3. **H,** Western blots of MYC in MDA-MB-231 and MDA-MB-468 cell lines after 72 hours of CHIR or BIO treatment. **I** and **J,** Flow cytometry of apoptotic (% Annexin V^+^) cells in MDA-MB-231-TGP and MDA-MB-468-TGP cell lines treated with DOC or CAR for 72 hours, with/without 48 hours CHIR or BIO pretreatment. One-way ANOVA and Dunnett correction, *n* = 3. Unless specified otherwise, all data are presented as the mean ± SEM. *, *P* < 0.05; **, *P* < 0.01; ***, *P* < 0.001; ****, *P* < 0.0001; ns, not significant.

To investigate the effects of activating the WNT signaling pathway in parental TNBC cells under chemotherapy-naïve conditions, we used two distinct GSK3 inhibitors, CHIR and BIO, which stabilize β-catenin (38). Treatment with either CHIR or BIO resulted in activation of the WNT signaling pathway (Fig. 3A and B) alongside a significant increase in transcriptional levels of WNT target gene, *AXIN2* (Supplementary Fig. S4A).

GSK3 inhibition significantly inhibited the proliferation of MDA-MB-231 cells, as observed with cell counts recorded over a 96-hour time-course (Fig. 3C). CHIR- and BIO-treated cells exhibited a significant decrease in growth rate at 96 hours (μ: 0.61 and 0.44 for CHIR and BIO, respectively; Fig. 3D) and significant increase in doubling time at 96 hours (27.2 and 40.4 hours for CHIR and BIO, respectively; Fig. 3E) compared with UNT cells (μ: 0.77 and doubling time = 21.66 hours). These findings were corroborated by additional testing using the MDA-MB-468 cell line (Supplementary Fig. S4B–S4D). Notably, the decline in cell count was not attributed to apoptotic effects exerted via either GSK3 inhibitor (Supplementary Fig. S4E). Immunofluorescence staining of proliferation marker Ki-67 highlighted a significant induction of growth arrest under WNT-stimulatory conditions (Fig. 3F), suggesting that activation of the WNT signaling pathway prompts a state of paused proliferation in TNBC cell lines.

CHIR or BIO treatment significantly reduced *MYC* and *NMYC* expression and MYC protein levels in MDA-MB-231 and MDA-MB-468 cells (Fig. 3G and H; Supplementary Fig. S4F), linking WNT activation to a suppressed MYC phenotype. To test whether WNT activation induces drug tolerance, TNBC cells were pretreated with CHIR or BIO, followed by chemotherapy. Pretreated cells showed reduced apoptosis compared with chemotherapy treatment alone (Fig. 3I and J; Supplementary Fig. S4G).

Together, these findings indicate that WNT activation in TNBC promotes growth arrest, downregulates MYC, and enhances chemoresistance, recapitulating key features of a diapause-like persister phenotype.

### Induction of transient *de novo* WNT signaling transcriptional activation in response to chemotherapy in *in vitro* TNBC cell lines and an *in vivo* TNBC xenograft model

The evolution of DTP cells during treatment remains a hotly debated topic, with some studies suggesting the enrichment of pre-existing DTP populations (16, 39, 40), whereas others propose a temporary phenotypic transition due to cellular plasticity (1, 2, 17, 18, 41). To explore the dynamics of this process, we monitored the activation of the WNT reporter in the WNT^High^ population through live-cell imaging.

**Figure 4.**
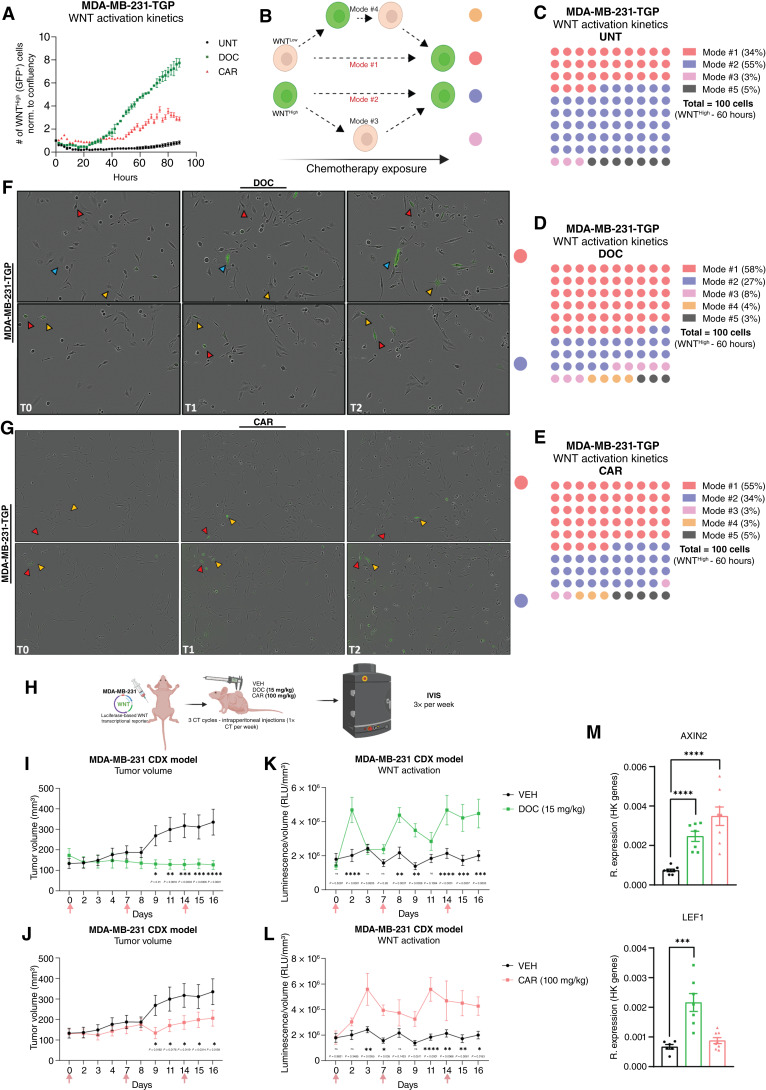
Induction of transient *de novo* WNT signaling transcriptional activation in response to chemotherapy in in *vitro* TNBC cell lines and in an *in vivo* TNBC xenograft model. **A,** Number of WNT^High^ (GFP^+^) cells by live-cell imaging, normalized to confluency, in MDA-MB-231-TGP cell line treated with DOC or CAR. **B,** Schematic of WNT activation fluctuation dynamics (mode-numbered and color-coded). **C–E,** Quantification of WNT activation dynamics in the MDA-MB-231-TGP cell line (UNT, DOC, or CAR). *n* = 100 cells tracked every 2 hours for 60 hours; each circle equals one cell, color-coded as in **B**. **F** and **G,** Time-lapse stills of MDA-MB-231-TGP cell line treated with DOC (top) or CAR (bottom) showing mode #1 and mode #2 (color-coded as in **B**). T0 = 0 hour; T1 = 30 hours; and T2 ≈ 50 hours. Colored arrows track the same cells across frames (horizontal). **H,** Cell line–derived xenograft experimental setup to study WNT signaling pathway kinetics under chemotherapy *in vivo*. MDA-MB-231 cells were engineered with the WNT transcriptional reporter TOPFLASH (33), a β-catenin–responsive firefly luciferase reporter plasmid, compatible for use with the *in vivo* live imaging system (IVIS). **I** and **J,** Tumor growth of subcutaneous xenografts treated with VEH, DOC (15 mg/kg/week; top), or CAR (100 mg/kg/week; bottom). Pink arrows, chemotherapy administration. Two-way ANOVA and Fisher least significant difference test, *n* = 8–7 mice per treatment group. **K** and **L,** Levels of WNT activation (RLU/mm^3^) displayed as luminescent signals (RLU) captured by IVIS Spectrum normalized to tumor volume (mm^3^) in xenografts treated with VEH, DOC (top), or CAR (bottom). Two-way ANOVA and Fisher least significant difference test, *n* = 8–7 mice per treatment group. **M,** RT-qPCR of *AXIN2* and *LEF1* (WNT target genes) in samples resected from xenograft models treated with VEH, DOC, or CAR, displayed as 2^−dCt^ [relative to housekeeping (HK) genes]. Unpaired *t* tests, *n* = 8–7 mice per treatment group. Unless specified otherwise, all data are presented as the mean ± SEM. *, *P* < 0.05; **, *P* < 0.01; ***, *P* < 0.001; ****, *P* < 0.0001; ns, not significant. **B,** Created in BioRender. Lluis Vinas, F. (2025) https://BioRender.com/xst2v9g; **H,** Created in BioRender. Lluis Vinas, F. (2025) https://BioRender.com/02xhwbh.

Under UNT conditions, levels of WNT^High^ cells remained stable (Fig. 4A, black line). In contrast, DOC or CAR treatment gradually enriched WNT^High^ cells (Fig. 4A, green and red lines), consistent with our previous FACS-based results. To visualize WNT transcriptional activation dynamics at single-cell resolution, we tracked the original WNT state of WNT^High^ cells (starting at 60 hours and back to 0 hour) under UNT or chemotherapy-treated conditions, defining distinct WNT activation dynamics (Fig. 4B and Supplementary representative images Supplementary Fig. S5A). In UNT conditions, 55% of WNT^High^ cells observed at 60 hours were initially WNT^High^ at T_0_, whereas 34% were activated during the culture span (modes #2 and #1, respectively; Fig. 4C, Supplementary representative images Supplementary Fig. S5B, and Supplementary Videos SV1 and SV2). Under DOC or CAR treatment, most WNT^High^ cells at 60 hours (58% and 55%, respectively) were *de novo*–activated from WNT^Low^ cells at T_0_ (mode #1), whereas only 27% and 34% were initially WNT^High^ at T_0_ (mode #2; Fig. 4D and E, representative images Fig. 4F and G, and Supplementary Videos SV3–SV6). These findings suggest that chemotherapy-induced WNT pathway enrichment primarily arises from *de novo* activation rather than selection of preexisting WNT^High^ cells. Additional dynamics (modes #3 and #4) occurred in minority cases, whereas cells that fell out of the imaging frame were considered of unknown origin/state (mode #5). Similar patterns were validated using the PDC-BRC-101-TGP cell line (Supplementary Fig. S5C–S5F).

Next, we assessed the dynamics after treatment was halted (Supplementary Fig. S5G). After chemotherapy withdrawal, diapause-like WNT^High^ fractions stabilized or increased for up to 3 weeks but declined after extended chemotherapy-free culture (4 weeks), restoring WNT populations to levels similar to that of UNT/basal-cultured cells (Supplementary Fig. S5H–S5K). Chemotherapy-recovered MDA-MB-231-TGP cell lines (DOC^REC^ and CAR^REC^) also reverted to normal (relative to UNT) rates of proliferation and drug tolerance (Supplementary Fig. S5L and S5M), confirming the transient and reversible nature of the diapause-like early persister phenotype driven by chemotherapy-induced WNT activation.

We next established cell line–derived xenograft models using MDA-MB-231 cells carrying a WNT transcriptional reporter (TOPFLASH; ref. 33) to examine *in vivo* dynamics (Fig. 4H). DOC or CAR treatment reduced tumor volume compared with VEH controls (Fig. 4I and J), without affecting body weight (Supplementary Fig. S5N; refs. 25, 42). IVIS imaging revealed WNT activation as early as 48 hours (DOC) or 72 hours (CAR), followed by a decline later in the week; this dynamic pattern repeated with subsequent doses (Fig. 4K and L, and Supplementary representative images Supplementary Fig. S5O). Gene expression analysis on the resected tumors confirmed elevated expression levels of WNT target genes (*AXIN2* and *LEF1*) in chemotherapy-treated groups (Fig. 4M).

Altogether, our *in vitro* findings show that the diapause-like WNT^High^ phenotype results namely from a *de novo* chemotherapy-driven action rather than solely representing a manifestation of an inherently chemotherapy-resistant subpopulation selected under treatment pressure. Notably, upon chemotherapy removal, WNT activity levels revert to baseline levels, indicating a transient enrichment of a diapause-like WNT^High^ cell state dependent on chemotherapy pressure. Furthermore, we demonstrate the activation dynamics of the WNT signaling pathway in chemotherapy-treated tumors within an *in vivo* setting, highlighting a transient and dynamic nature.

### Chemotherapeutic treatment induces elevated transcriptional expression of WNT ligands, WNT enhancers, and WNT secretion machinery components

**Figure 5.**
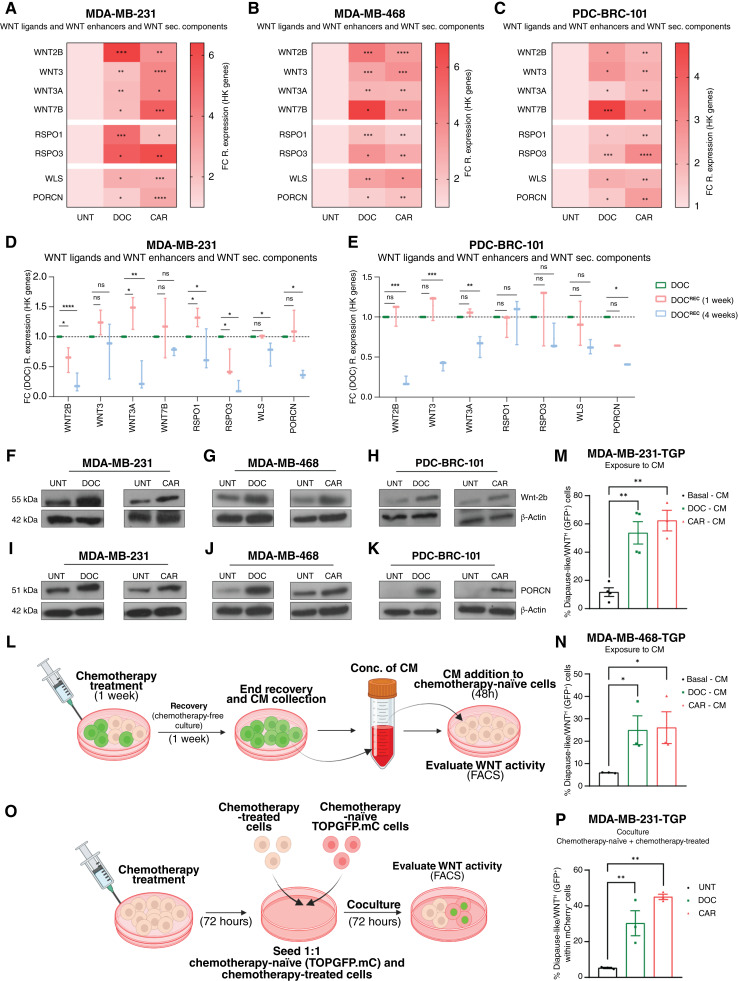
Chemotherapeutic treatment induces elevated transcriptional expression of WNT ligands, WNT enhancers, and WNT secretion machinery components. **A–C,** qRT-PCR heatmaps of WNT ligands (*WNT2B*, *WNT3*, *WNT3A*, and *WNT7B*), WNT enhancers (*RSPO1* and *RSPO3*), and WNT ligand secretion machinery components (*WLS* and *PORCN*) in TNBC cell lines treated with DOC or CAR for 72 hours, displayed as FC (to UNT) of 2^−dCt^ [relative to housekeeping (HK) genes]. Unpaired *t* tests on 2^−dCt^ values, *n* = 3. **D** and **E,** qRT-PCR of same genes as in **A–C** in MDA-MB-231 and PDC-BRC-101 cell lines treated with DOC following the scheme in Supplementary Fig. S5G, displayed as FC (to DOC) of 2^−dCt^ values (relative to HK genes). Unpaired *t* tests on 2^−dCt^ values, *n* = 3. **F–H,** Western blots of Wnt-2b in TNBC cell lines treated with DOC or CAR for 72 hours. **I–K,** Western blots of PORCN in TNBC cell lines treated with DOC or CAR for 72 hours. **L,** CM experimental setup. **M** and **N,** Flow cytometry of % diapause-like/WNT^High^ (GFP^+^) cells within viable (DAPI^−^) MDA-MB-231-TGP and MDA-MB-468-TGP cell lines cultured with concentrated basal-, DOC-, or CAR-CM for 48 hours. Unpaired *t* tests, *n* = 3. **O,** Coculture experimental setup. **P,** Flow cytometry of % diapause-like/WNT^High^ (GFP^+^) cells within viable (DAPI^−^) chemotherapy-naïve MDA-MB-231-TGP.mC (mCherry-TOP-GFP) cocultured with DOC- or CAR-treated MDA-MB-231 cells for 72 hours. Unpaired *t* tests, *n* = 3. Unless specified otherwise, all data are presented as the mean ± SEM. *, *P* < 0.05; **, *P* < 0.01; ***, *P* < 0.001; ****, *P* < 0.0001; ns, not significant. **L,** Created in BioRender. Lluis Vinas, F. (2025) https://BioRender.com/j9nb1n9; **O,** Created in BioRender. Lluis Vinas, F. (2025) https://BioRender.com/j9nb1n9.

The WNT signaling pathway is activated by 19 extracellular WNT ligands binding membrane receptors, with secretion requiring Porcupine (PORCN)-mediated acylation and transport by Wntless/evenness interrupted (31, 43). Additionally, the RSPO protein family has been shown to enhance WNT ligand activity to further promote WNT pathway activation (31, 43). Focusing on established canonical WNT and RSPO ligands, we found *WNT2B*, *WNT3*, *WNT3A*, *WNT7B*, *RSPO1*, and *RSPO3*, as well as WNT ligand secretion machinery, *WLS* and *PORCN*, to be steadily expressed in basal conditions across all analyzed TNBC cell lines (Supplementary Fig. S6A–S6C) and significantly increased under chemotherapy-treatment conditions (Fig. 5A–C), suggesting that chemotherapeutic exposure actively promotes elevated transcription levels of several key components involved in canonical WNT pathway activation.

Furthermore, gene expression analysis performed on the resected tumors from our previous *in vivo* experimental setup (Fig. 4H) revealed that expression of various WNT ligands (*WNT2B*, *WNT3A*, and *WNT7B*), enhancers (*RSPO1* and *RSPO3*), and secretion machinery components (*WLS* and *PORCN*) was upregulated in chemotherapy-treated groups (Supplementary Fig. S6D).

During drug holidays, the expression levels of the majority of WNT activation components remained elevated after 1 week of chemotherapy recovery (Fig. 5D and E, pink bars) but declined after 4 weeks, coinciding with the return of diapause-like WNT^High^ cells to basal levels (Fig. 5D and E, light blue bars, and Supplementary Fig. S5H–S5K) and indicating that exit from a diapause-like early persister cell state correlates with decreased WNT ligand expression upon drug holidays. Western blots confirmed upregulation of the WNT ligand Wnt-2b and the acyltransferase PORCN after DOC or CAR treatment in all TNBC lines (Fig. 5F–K).

CM from 1-week chemotherapy recovery cultures increased diapause-like WNT^High^ cells in chemotherapy-naïve populations (CM, Fig. 5L–N). Likewise, coculture of chemotherapy-naïve (MDA-MB-231-mCherry-TGP) with chemotherapy-recovering (MDA-MB-231) cells significantly enriched WNT^High^ cells in the naïve population (Fig. 5O and P).

In summary, our findings demonstrate that chemotherapeutic treatment leads to elevated expression levels of WNT ligands, enhancers, and components of the WNT secretory apparatus, highlighting a significant role for WNT activators in regulating entry into and exit from a diapause-like WNT^High^ state under chemotherapeutic pressure.

### WNT ligand secretion inhibition alongside chemotherapeutic treatment hinders diapause-like early persister cell enrichment *in vitro* and synergistically sensitizes an *in vivo* TNBC xenograft model

**Figure 6.**
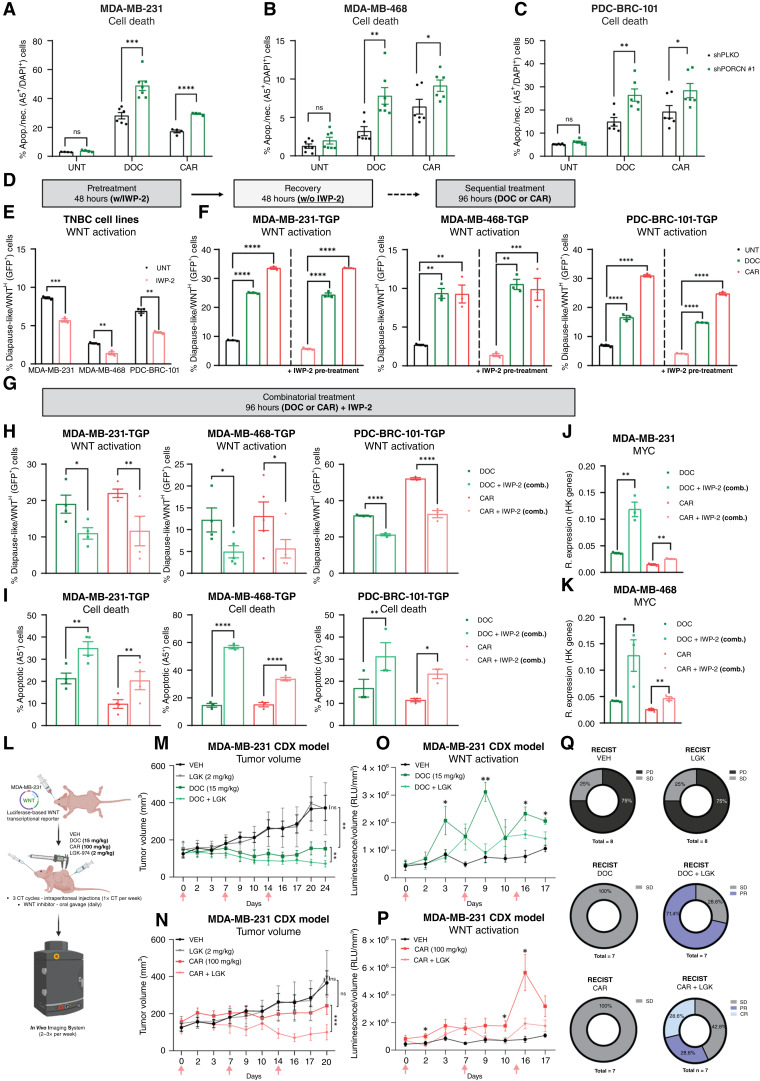
WNT ligand secretion inhibition alongside chemotherapeutic treatment hinders diapause-like early persister cell enrichment *in vitro* and synergistically sensitizes an in *vivo* TNBC xenograft model. **A–C,** Flow cytometry of apoptotic and necrotic (% Annexin V^+^/DAPI^+^) cells in TNBC cell lines (shPLKO vs. shPORCN#1) treated with DOC or CAR for 96 hours. Unpaired *t* tests, *n* = 4. **D,** Sequential treatment model. IWP-2 pretreatment (48 hours) followed by a recovery period then chemotherapy. **E,** Flow cytometry of % diapause-like/WNT^High^ (GFP^+^) within viable (DAPI^−^) cells in TNBC-TGP cell lines pretreated with IWP-2 (10 μmol/L, 48 hours). Multiple *t* tests, Holm–Sidak correction, *n* = 3. **F,** Flow cytometry of % diapause-like/WNT^High^ (GFP^+^) within viable (DAPI^−^) cells in TNBC-TGP cell lines treated with DOC or CAR for 96 hours (with or without IWP-2 pretreatment). Multiple *t* tests, Holm–Sidak correction, *n* = 3. **G,** Combinatorial treatment model schematic. **H,** Flow cytometry of % diapause-like/WNT^High^ (GFP^+^) within viable (DAPI^−^) cells in TNBC-TGP cell lines treated with DOC or CAR for 96 hours (sole or in combination with IWP-2). Unpaired t tests, *n* = 4. **I,** Flow cytometry of apoptotic (% Annexin V^+^) cells in TNBC cell lines treated with DOC or CAR for 96 hours (sole or in combination with IWP-2). Unpaired *t* tests, *n* = 4. **J** and **K,** qRT-PCR of *MYC* in MDA-MB-231 and MDA-MB-468 cell lines treated with DOC or CAR for 96 hours (sole or in combination with IWP-2), displayed as 2^−dCt^ [relative to housekeeping (HK) genes]. Unpaired *t* tests, *n* = 3. **L,** Cell line–derived xenograft experimental setup with WNT ligand secretion inhibition *in vivo*. **M** and **N,** Tumor growth of subcutaneous xenografts treated with VEH, LGK (2 mg/kg/day), DOC, DOC + LGK (top), CAR, or CAR + LGK (bottom). Pink arrows, chemotherapy administration. Paired *t* tests (based on all tumor volumes), *n* = 8–5 mice per treatment group. **O** and **P,** Levels of WNT activation (RLU/mm^3^) captured by IVIS Spectrum in xenografts treated as in **M** and **N**. Multiple *t* tests, *n* = 8–5 mice per treatment group. For **M–P**, the number of animals was as follows: VEH = 8, LGK = 7, DOC = 7, DOC + LGK = 6, CAR = 7, and CAR + LGK = 5 animals. **Q,** RECIST classification (% animals per group) as complete response (CR), partial response (PR), stable disease (SD), or progressive disease (PD) for VEH, LGK, DOC, DOC + LGK, CAR, and CAR + LGK. Unless specified otherwise, all data are presented as the mean ± SEM. *, *P* < 0.05; **, *P* < 0.01; ***, *P* < 0.001; ****, *P* < 0.0001; ns, not significant. **L,** Created in BioRender. Lluis Vinas, F. (2025) https://BioRender.com/02xhwbh.

Previous work linked therapy-induced WNT activation to cell-autonomous mechanisms independent of ligand secretion (44). To test whether diapause-like persister formation depends on WNT ligand secretion, we stably transduced TNBC cells with lentiviral shRNAs against PORCN, which is required for WNT ligand secretion (45). Chemotherapy-treated PORCN-silenced cells (shPORCN#1) showed reduced active β-catenin and fewer diapause-like WNT^High^ cells, confirming an essential role for PORCN in chemotherapy-induced enrichment (Supplementary Fig. S7A–S7C). Whereas PORCN silencing did not affect viability under basal conditions, it markedly increased apoptosis and necrosis upon chemotherapy, indicating strong sensitization (Fig. 6A–C). Similar results were obtained with a second shRNA (shPORCN#4; Supplementary Fig. S7D–S7F).

Next, we investigated whether pharmacologic inhibition of PORCN could also prove effective in curbing the induction of diapause-like WNT^High^ cells under treatment pressure. We examined two distinct approaches to target treatment-induced diapause-like WNT^High^ cells. In the first approach, we pretreated TNBC cell lines with the inhibitor of WNT production-2 (IWP-2; ref. 46) for 48 hours, followed by the application of chemotherapy (sequential treatment). In the second approach, we applied chemotherapeutic treatment simultaneously in combination with IWP-2 (combinatorial treatment).

Pretreatment (sequential treatment strategy; Fig. 6D) with IWP-2 led to a notable reduction in the percentage of diapause-like WNT^High^ cells (Fig. 6E) with no effects on cell viability (Supplementary Fig. S7G–S7I, black and gray bars). However, subsequent chemotherapy treatment induced robust WNT activation similar to chemotherapy treatment alone (Fig. 6F), and no sensitization effect was observed (Supplementary Fig. S7G–S7I), indicating that sequential inhibition cannot prevent *de novo* chemotherapy-induced diapause-like WNT^High^ cell enrichment.

In the second approach (combinatorial treatment strategy; Fig. 6G), simultaneous treatment with chemotherapy in combination with IWP-2 led to a significant decrease in the percentage of diapause-like WNT^High^ cells (Fig. 6H) and resulted in a substantial increase in apoptotic cell death compared with treatment with either chemotherapeutic agent alone (Fig. 6I). Interestingly, we observed that the supplementation of IWP-2 alongside chemotherapy resulted in a significant rescue of *MYC* levels (Fig. 6J and K). Similar results were obtained with a second PORCN inhibitor, LGK-974 (WNT-974; Supplementary Fig. S7J–S7O; refs. 25, 47, 48).

Interestingly, the use of LGK-974 in *in vivo* xenograft models (Fig. 6L), combined with either DOC or CAR treatment, resulted in a substantial and significant decrease in WNT pathway activation, correlating with marked reduction in tumor volume compared with sole chemotherapy-, LGK-, or VEH-treated groups (Fig. 6M–P, representative images Supplementary Fig. S7P, and Supplementary Table S7). Notably, LGK treatment, alone or in combination with chemotherapy, had no effect on mouse body weight observed during the treatment course (Supplementary Fig. S7Q; ref. 49).

RECIST (50) analysis was performed to assess tumor response to treatment, categorizing outcomes into PD, SD, PR, and CR. In the VEH group, 75% (6/8) of tumors were PD and 25% (2/8) were SD (Fig. 6Q). LGK-974 alone produced a similar pattern (75% PD and 25% SD), indicating no impact on tumor response. Sole DOC and CAR treatment yielded 100% (7/7) SD, confirming chemotherapy efficacy in controlling tumor growth. In the DOC + LGK group, 28.6% (2/7) tumors were SD and 71.4% (5/7) were PR, improving outcomes compared with DOC alone (100% SD). Similarly, in the CAR + LGK group, 42.8% (3/7) were SD, 28.6% (2/7) PR, and 28.6% (2/7) CR, demonstrating enhanced responses with combination therapy (Fig. 6Q).

In summary, simultaneous, rather than sequential, treatment with chemotherapy and PORCN inhibitors reduced diapause-like WNT^High^ enrichment *in vitro* and significantly enhanced TNBC tumor sensitivity to chemotherapy *in vivo*.

### Preclinical PDO models recapitulate chemotherapy-mediated WNT activation and sensitization to synergistic WNT ligand secretion inhibition

**Figure 7.**
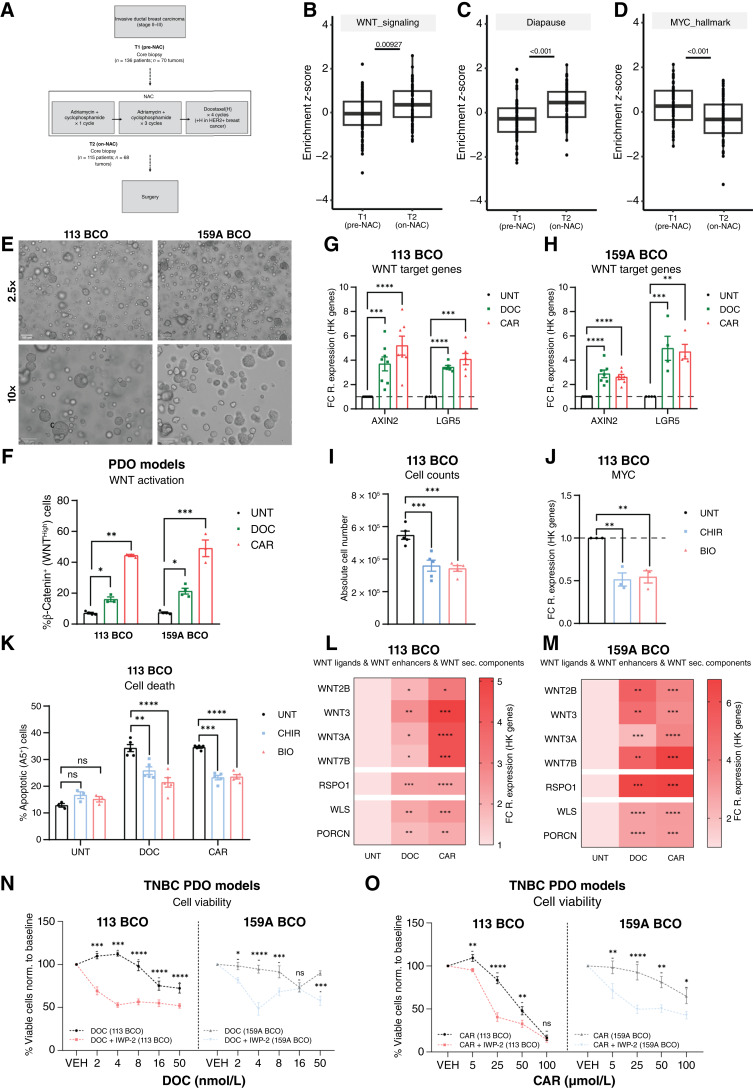
Preclinical PDO models recapitulate chemotherapy-mediated WNT activation and sensitization to synergistic WNT ligand secretion inhibition. **A,** Overview of patient dataset (GSE123845) analyzed in this study. **B–D,** Boxplots showing single-sample gene set enrichment scores of our in-house derived WNT^High^ signature, a diapause-DTP signature (1), and MYC gene set enrichment analysis hallmark signature in RNA-seq data of breast cancer biopsies obtained prior to (pre-NAC; *n* = 70 tumors) and during NAC (on-NAC; *n* = 68 tumors). **E,** Phase-contrast images of TNBC-PDO models 113 BCO (left) and 159A BCO (right) under basal conditions at 2.5× (top) and 10× (bottom) magnification. **F,** Flow cytometry of % β-catenin^+^ WNT-active cells in 113 BCO and 159A BCO models treated with DOC (16 nmol/L for 113 BCO and 8 nmol/L for 159A BCO) or CAR (50 μmol/L for 113 BCO and 125 μmol/L for 159A BCO) for 96 hours. Multiple *t* tests, Holm–Sidak correction, *n* = 3. **G** and **H,** qRT-PCR of WNT target genes (*AXIN2* and *LGR5*) in 113 BCO and 159A BCO models treated with DOC or CAR for 96 hours, displayed as FC (to UNT) of 2^−dCt^ [relative to housekeeping (HK) genes]. Multiple *t* tests on 2^−dCt^ values, Holm–Sidak correction, *n* = 4. **I,** Absolute cell number of 113 BCO model treated with CHIR (8 μmol/L) or BIO (3 μmol/L) for 72 hours. Unpaired *t* tests, *n* = 3. **J,** RT-qPCR of *MYC* in 113 BCO model treated with CHIR or BIO for 72 hours, displayed as FC (to UNT) of 2^−dCt^ (relative to HK genes). Unpaired *t* tests on 2^−dCt^ values, *n* = 3. **K,** Flow cytometry of apoptotic (% Annexin V^+^) cells in 113 BCO model treated with DOC or CAR for 96 hours (sole or pretreated with CHIR or BIO for 48 hours). Two-way ANOVA and Tukey correction, *n* = 3. **L** and **M,** qRT-PCR heatmaps of WNT ligands, enhancers, and secretion machinery components in 113 BCO and 159A BCO models treated with DOC or CAR for 96 hours, displayed as FC (to UNT) of 2^−dCt^. Unpaired *t* tests on 2^−dCt^ values, *n* = 4. **N** and **O,** Drug dose–response curves of 113 BCO and 159A BCO models treated with DOC or CAR (sole or in combination with IWP-2, 50 μmol/L). Viability in sole or combinatorial treatment is normalized to UNT or sole IWP-2 conditions (baseline). Multiple *t* tests, Holm–Sidak correction, *n* = 4. Unless specified otherwise, all data are presented as the mean ± SEM. *, *P* < 0.05; **, *P* < 0.01; ***, *P* < 0.001; ****, *P* < 0.0001; ns, not significant. **A,** Created in BioRender. Lluis Vinas, F. (2025) https://BioRender.com/xst2v9g.

Transcriptomic analysis of longitudinally paired breast cancer patient samples during NAC treatment (GSE123845; Fig. 7A; ref. 51) showed that a WNT signaling signature (derived from chemotherapy-sorted WNT^High^ cells) was significantly enriched in tumor samples obtained from patients undergoing (on-NAC) NAC treatment (Fig. 7B; Supplementary Table S8). Similarly, a significant enrichment of the Rehman and colleagues (1) diapause-like gene signature and a significant reduction in the MYC hallmark signature (Fig. 7C and D) were seen in samples obtained from patients on-NAC, further highlighting the interplay between the WNT^High^ and diapause-like cell phenotypes in a clinical setting.

Next, we investigated the effects of chemotherapy on preclinical 3D PDO models (52). Two models, R1-IDC113 [113 breast cancer organoid (BCO)] and R2-IDC159A (159A BCO; Fig. 7E; Supplementary Fig. S8A), were used. Organoid models are typically cultured in growth factor–rich medium (53), including WNT ligand (Wnt-3a) and WNT ligand enhancer (RSPO3), possibly influencing studies of WNT pathway dynamics. Culturing either organoid model for four passages in a WNT^−^/RSPO^−^ BCO medium had no effect on the morphology, proliferation rate, or viability when compared with a baseline (WNT^+^/RSPO^+^) BCO medium (Supplementary Fig. S8B and S8C; ref. 54). Upon exposure to IC_50_ concentrations of either DOC or CAR (Supplementary Fig. S8D–S8G) in a WNT^−^/RSPO^−^ BCO medium, both PDO models exhibited a significant increase in active β-catenin levels (Fig. 7F; Supplementary Fig. S8H) and in expression levels of WNT target genes (Fig. 7G and H).

In the 113 BCO model, CHIR or BIO treatment resulted in transcriptional activation of WNT signaling as evidenced by increased WNT target gene expression (*AXIN2*; Supplementary Fig. S8I). In parallel, treatment with CHIR or BIO led to a significant decrease in cell number as a possible consequence of growth arrest (Fig. 7I) seen in conjunction with suppression of *MYC* and *NMYC* (Fig. 7J; Supplementary Fig. S8J). Furthermore, pretreatment with either CHIR or BIO induced a drug-tolerant phenotype, whereby we observed a significant decrease in apoptosis induction after chemotherapeutic treatment when compared with sole chemotherapy exposure (Fig. 7K).

Next, we confirmed significant elevation in the expression levels of WNT ligands, enhancers, and secretion machinery components in chemotherapy-treated PDO models (Fig. 7L and M). To investigate the efficacy of the combinatorial treatment strategy, both PDO models were exposed to WNT ligand secretion inhibition alone and in combination with increasing concentrations of either chemotherapeutic agent for 96 hours. At the used concentration, treatment of both PDO models with IWP-2 alone did not have any effect on cell viability or proliferation (Supplementary Fig. S8K), in accordance with the WNT^−^/RSPO^−^ culture conditions. However, exposure of both PDO models to chemotherapy in combination with IWP-2 led to a significant reduction in cell viability compared with chemotherapy alone (Fig. 7N and O). Interestingly, this sensitization effect was most pronounced when IWP-2 was supplemented with sublethal concentrations of chemotherapy (<16 nmol/L DOC and <50 μmol/L CAR – 113 BCO | <8 nmol/L DOC and <125 μmol/L CAR – 159A BCO).

In summary, our data demonstrate chemotherapy-induced WNT activation in TNBC preclinical PDO models and in transcriptomic datasets derived from on-NAC patients’ samples. PDO models exhibited a robust and enhanced sensitization to the combinatorial treatment approach comprising WNT ligand secretion inhibition alongside sublethal (<determined IC_50_) concentrations of chemotherapy.

## Discussion

Recent studies show that persister cells adopt a slow-growing state resembling dormancy or embryonic diapause, marked by negative *MYC* activity (1, 2). Whereas several nongenetic mechanisms promote drug tolerance (5, 25, 55, 56), the drivers of a diapause-like persister state remain unclear. Understanding the early events leading to this reversible state may reveal strategies to prevent drug resistance before it becomes established. Persistence typically emerges after approximately 9 days of continuous treatment *in vitro* (19), but the molecular cues initiating diapause-like features are poorly defined. In this study, we identified 72 hours as an early response point, with ∼50% fewer cells and ∼30% reduced viability, marking the onset of persister development. We show that WNT pathway activation not only induces but also serves as a biomarker of early diapause-like phenotypes, particularly under chemotherapy, but also in UNT parental cells. Moreover, we show that among early persister cells, only WNT-active cells acquire genuine transcriptional and functional diapause-like features.

Although mutations in WNT pathway components are rare in TNBC, dysregulated canonical WNT signaling and β-catenin stabilization have been linked to poor prognosis (31, 57–60). Prior studies mainly examined WNT in TNBC under unchallenged conditions (61, 62), whereas our work focuses on responses during chemotherapy. We found significant enrichment of WNT activity (WNT^High^ population) in treated tumors, indicating that even cancers with initially low or normal WNT levels can acquire WNT enrichment under therapy, promoting a diapause-like persister state. Current trials (NCT03447470 and NCT01351103) testing PORCN inhibition are limited to WNT-deregulated cancers and do not consider chemotherapy (47, 48). Our results indicate that inhibiting WNT ligand secretion could be beneficial not only for tumors initially characterized as WNT-addicted under baseline conditions but also for all patients with TNBC undergoing chemotherapy, whereby WNT ligand secretion inhibition would prevent the enrichment of a diapause-like persister cell population.

Wang and colleagues (44) recently showed that Otulin, a linear linkage-specific deubiquitinase, enhances β-catenin activity in a WNT ligand–independent manner and under genotoxic stress, primarily within the first 24 hours of drug treatment. In contrast, our study demonstrates that chemotherapy-induced WNT activation in TNBC depends on WNT ligands and persists well beyond this early window. Traditionally, WNT activation is linked to (i) increased proliferation through *MYC* upregulation (36, 37, 63, 64) and (ii) BCSC maintenance promoting drug tolerance (25, 57–59, 65). However, we find that chemotherapy-induced WNT activation does not enrich BCSCs (CD24^Low^/CD44^High^) but instead promotes a reversible diapause-like persister state. Moreover, WNT activation—via chemotherapy or GSK3 inhibition—drives a slow-proliferating, *MYC*-low transcriptional state resembling diapause. Prior reports confirm that, in certain contexts, WNT can suppress MYC (66–68). One possible explanation for this context-dependent effect is the complex interplay between WNT signaling and other pathways, such as TGFβ. Notably, WNT activation can induce TGFβ signaling (69, 70), which is known to antagonize *MYC* expression by promoting cell quiescence and differentiation, ultimately reducing *MYC* transcription (71, 72). Notably, reduced proliferation upon WNT activation in TNBC parallels findings in LGR5^+^ basal cell carcinoma models, in which therapy-induced WNT activity drives a slow-proliferative phenotype (73). This suggests that WNT activation may also promote diapause-like states in other cancers.

Enrichment of DTP cells has been observed across various chemotherapeutic treatments (3–5, 19). In this study, we examined DOC, which stabilizes microtubules, and CAR, which induces DNA crosslinks. Despite their different proapoptotic mechanisms, both treatments enriched diapause-like WNT^High^ cells, showing that distinct chemotherapies converge on WNT ligand upregulation and pathway activation. Interestingly, in regenerative models, such as *Hydra*, increased WNT ligand expression has been noted in cells undergoing apoptosis as a prosurvival mechanism in response to tissue damage (74). This suggests that, under chemotherapy, cells may activate analogous prosurvival mechanisms. Interestingly, our RNA-seq further revealed enrichment of other developmental and oncogenic pathways, including Hedgehog, Notch, IL6/JAK/STAT3, and TGFβ, in both chemotherapy-naïve and treated WNT^High^ cells, highlighting the possible involvement of additional cascades in persister induction.

Most current strategies target established DTP cells, for example, with BET inhibitors (75), rather than preventing the emergence of a diapause-like persister state. Our results show that combining WNT ligand secretion inhibitors with chemotherapy reduces diapause-like WNT^High^ enrichment and sensitizes tumors, highlighting translational potential for TNBC treatment. Importantly, timing is critical: pretreatment with PORCN inhibitors did not block the subsequent rise of WNT^High^ cells during chemotherapy, indicating limited benefit for sequential regimens. Instead, our findings support simultaneous combinatorial treatment with WNT inhibitors and chemotherapy to prevent diapause-like enrichment while enhancing tumor sensitivity.

The origin of DTP cells remains debated: some studies suggest a stable clonal origin, whereas others propose a transient drug-tolerant state induced by chemotherapy. Our live-cell imaging studies, which allow the tracking and tracing of WNT reporter TNBC cell lines, indicate that chemotherapy treatment leads to a significant enrichment of diapause-like WNT^High^ phenotype, primarily in cells that were initially in a WNT^Low^ state. This suggests a vital role of *de novo* activation of the WNT signaling pathway in response to chemotherapeutic treatment. Additionally, a lower but notable proportion of diapause-like WNT^High^ cells in chemotherapy-treated conditions were initially in a transcriptionally WNT-active state. These observations imply that both intrinsic and acquired resistance mechanisms, driven by WNT transcriptional activity, coexist and contribute to early DTP cell formation. Notably, GSK3 inhibition alone induced a diapause-like state in parental TNBC cells, demonstrating that this phenotype can arise independently of therapy and explaining its presence in chemotherapy-naïve populations (3–5). Importantly, the diapause-like state is transient and reversible: cells exit during drug holidays in parallel with reduced WNT ligand expression, indicating that WNT signaling regulates both entry into and exit from the diapause-like state.

A limitation of our study is the inability to resolve WNT signaling at single-cell resolution in our *in vivo* experiments. Whereas our luciferase-based WNT reporter enables real-time, noninvasive imaging of WNT pathway activation in response to chemotherapy, it does not allow for cell sorting or detailed profiling of WNT^High^ versus WNT^Low^ cells after tumor resection. In contrast to our *in vitro* model—in which fluorescence-based sorting permits analysis of transcriptional and functional heterogeneity—our *in vivo* experimental setup is restricted to bulk-level luminescence. Consequently, we refrained from assigning WNT^High^ or WNT^Low^ designation to tumors *in vivo* and instead focused on pathway activation at the whole-tumor level. Furthermore, the proliferative nature of the tumors *in vivo* precludes classification of these cells as DTPs or diapause-like cells. These limitations reflect both technical constraints and the complexity of modeling early persister states *in vivo*, underscoring the need for future models capable of spatiotemporal resolution of WNT signaling at the single-cell level.

Our work highlights that the transcriptional activity of the WNT signaling pathway, in parental (chemotherapy-naïve) tumors as well as under chemotherapy-treatment conditions, serves as a biomarker and mechanistic player inducing a diapause-like persister cell state. A combinatorial treatment strategy using chemotherapeutic agents alongside targeting the WNT signaling pathway through WNT ligand secretion inhibition is able to significantly impact the enrichment and induction of a diapause-like persister cell population, significantly sensitizing tumor cells to therapy.

## Supplementary Material

Figure S1 SUP. Fig. 1 - Distnct chemotherapy treatments converge on robust WNT/β-catenin pathway activation during early persister cell enrichment

Figure S2 SUP. Fig. 2 - Distnct chemotherapy treatments converge on robust WNT/β-catenin pathway activation during early persister cell enrichment

Figure S3 SUP. Fig. 3 - Parental and early chemotherapy-treated WNTHigh persister cells display diapause-like cell properties.

Figure S4 SUP. Fig. 4 - WNT pathway activation triggers reduced proliferation mimicking a diapause-like state in parental TNBC cells

Figure S5 SUP. Fig. 5 - Induction of transient de novo WNT signaling transcriptional activation in response to chemotherapy in in vitro TNBC cell lines and in an in vivo TNBC xenograph model.

Figure S6 SUP. Fig. 6 - Chemotherapeutic treatment induces elevated transcriptional expression of WNT ligands, WNT enhancers, and WNT secretion machinery components

Figure S7 SUP. Fig. 7 - WNT ligand secretion-inhibition alongside chemotherapeutic treatment hinders diapause-like early persister cell enrichment in vitro and synergistically sensitizes an in vivo TNBC xenograph model

Figure S8 SUP. Fig. 8 - Preclinical PDO models recapitulate chemotherapy-mediated WNT activation and sensitization to synergistic WNT ligand secretion-inhibition

Supplementary Video 1 MDA-MB-231-TGP cell line in UNT conditions (WNT enrichment).

Supplementary Video 2 MDA-MB-231-TGP cell line in UNT conditions (De novo WNT activation).

Supplementary Video 3 MDA-MB-231-TGP cell line in DOC-treatment conditions (WNT enrichment).

Supplementary Video 4 MDA-MB-231-TGP cell line in DOC-treatment conditions (De novo WNT activation).

Supplementary Video 5 MDA-MB-231-TGP cell line in CAR-treatment conditions (WNT enrichment).

Supplementary Video 6 MDA-MB-231-TGP cell line in CAR-treatment conditions (De novo WNT activation).

Supplementary Table S1 Supplementary Table S1 - MTT-IC50 curves

Supplementary Table S2 Supplementary Table S2 - DESEQ

Supplementary Table S3 Supplementary Table S3 - GSEA

Supplementary Table S4 Supplementary Table S4 - DESEQ

Supplementary Table S5 Supplementary Table S5 - Regression model (Forrest Plots)

Supplementary Table S6 Supplementary Table S6 - Wilcox Plots

Supplementary Table S7 Supplementary Table S7 - Statistical Tests (Tumor Volumes)

Supplementary Table S8 Supplementary Table S8 - GSE123845

## Data Availability

The bulk mRNA-seq data generated in this study are publicly available in the GEO under accession number GSE254558 (https://www.ncbi.nlm.nih.gov/geo/query/acc.cgi?acc=GSE254558). Publicly available data analyzed in this study were obtained from GEO at accession number GSE123845 (https://www.ncbi.nlm.nih.gov/geo/query/acc.cgi?acc=GSE123845). The code(s) used in generating the data supporting the conclusions of this article is available in a Code Ocean repository at https://codeocean.com/capsule/9366404/tree/v1. All other raw data are available upon request to the corresponding author.
