# Supplementary material for: Chemotherapy-Treated Breast Cancer Cells Activate the WNT Signaling Pathway to Enter a Diapause-Like Early Persister State
Source: Cancer Res. 2025 Oct 21;86(2):310–30. doi: 10.1158/0008-5472.CAN-24-4165 (PMC12809118; doi:10.1158/0008-5472.CAN-24-4165)
Supplement: Figure S1 — SUP. Fig. 1 - Distnct chemotherapy treatments converge on robust WNT/β-catenin pathway activation during early persister cell enrichment [file can-24-4165_figure_s1_suppsf1.pdf]

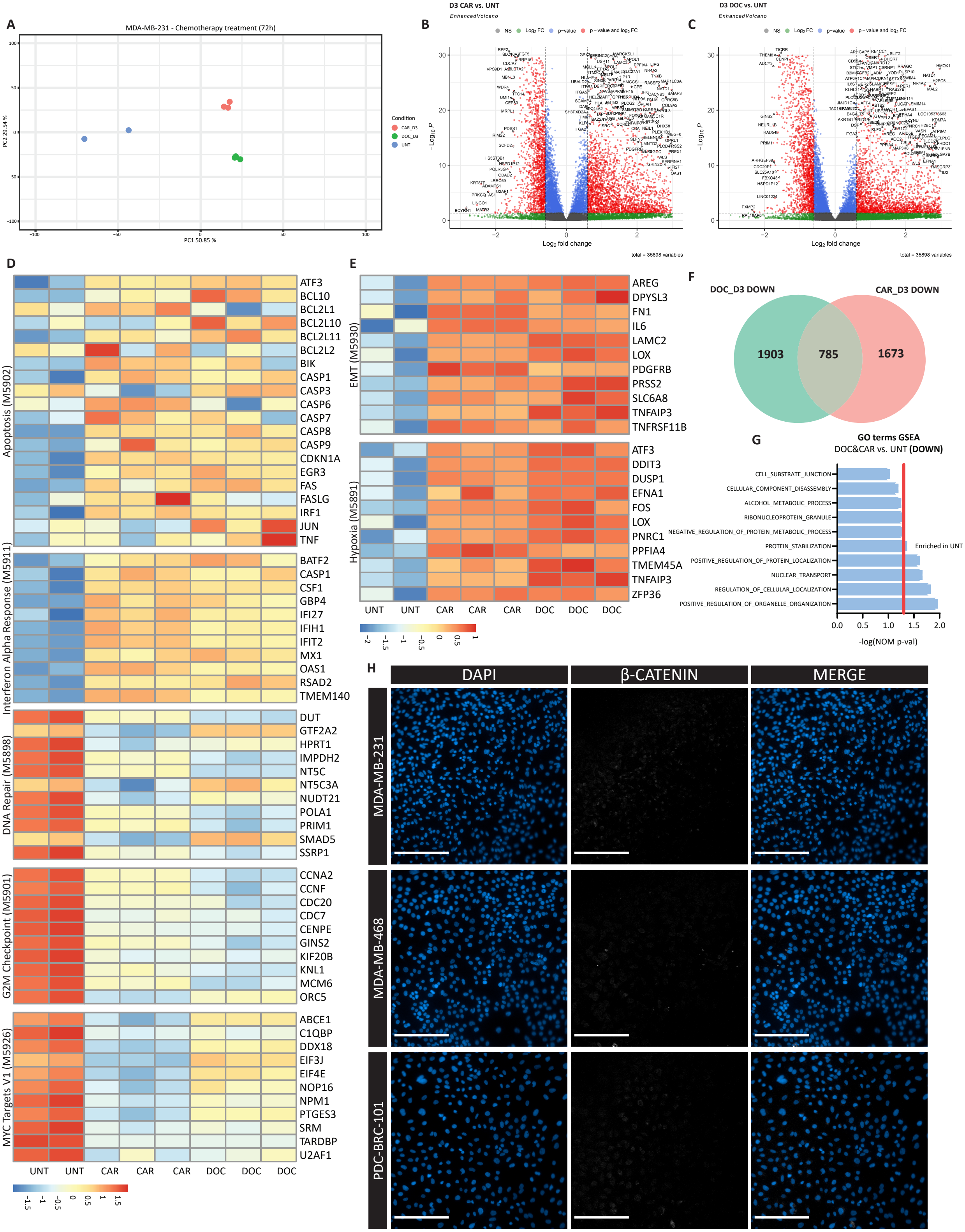

A
MDA-MB-231 - Chemotherapy treatment (72h)
PC1 50.85 %
PC2 29.54 %
Condition
CAR_D3
DOC_D3
UNT
B
D3 CAR vs. UNT
EnhancedVolcano
C
D3 DOC vs. UNT
EnhancedVolcano
NS
Log2 FC
p-value
p - value and log2 FC
Log2 fold change
total = 35898 variables
D
Apoptosis (M5902)
ATF3
BCL10
BCL2L1
BCL2L10
BCL2L11
BCL2L2
BIK
CASP1
CASP3
CASP6
CASP7
CASP8
CASP9
CDKN1A
EGR3
FAS
FASLG
IRF1
JUN
TNF
Interferon Alpha Response (M5911)
BATF2
CASP1
CSF1
GBP4
IFI27
IFIH1
IFIT2
MX1
OAS1
RSAD2
TMEM140
DNA Repair (M5898)
DUT
GTF2A2
HPRT1
IMPDH2
NT5C
NT5C3A
NUDT21
POLA1
PRIM1
SMAD5
SSRP1
G2M Checkpoint (M5901)
CCNA2
CCNF
CDC20
CDC7
CENPE
GINS2
KIF20B
KNL1
MCM6
ORC5
MYC Targets V1 (M5926)
ABCE1
C1QBP
DDX18
EIF3J
EIF4E
NOP16
NPM1
PTGES3
SRM
TARDBP
U2AF1
UNT UNT CAR CAR CAR DOC DOC DOC
E
EMT (M5930)
AREG
DPYSL3
FN1
IL6
LAMC2
LOX
PDGFRB
PRSS2
SLC6A8
TNFAIP3
TNFRSF11B
Hypoxia (M5891)
ATF3
DDIT3
DUSP1
EFNA1
FOS
LOX
PNRC1
PPFIA4
TMEM45A
TNFAIP3
ZFP36
UNT UNT CAR CAR CAR DOC DOC DOC
F
DOC_D3 DOWN
CAR_D3 DOWN
1903
785
1673
G
GO terms GSEA
DOC&CAR vs. UNT (DOWN)
CELL_SUBSTRATE_JUNCTION
CELLULAR_COMPONENT_DISASSEMBLY
ALCOHOL_METABOLIC_PROCESS
RIBONUCLEOPROTEIN_GRANULE
NEGATIVE_REGULATION_OF_PROTEIN_METABOLIC_PROCESS
PROTEIN_STABILIZATION
POSITIVE_REGULATION_OF_PROTEIN_LOCALIZATION
NUCLEAR_TRANSPORT
REGULATION_OF_CELLULAR_LOCALIZATION
POSITIVE_REGULATION_OF_ORGANELLE_ORGANIZATION
Enriched in UNT
-log(NOM p-val)
H
DAPI
β-CATENIN
MERGE
MDA-MB-231
MDA-MB-468
PDC-BRC-101

SUP. Fig. 1: Distinct chemotherapy treatments converge on robust WNT/β-catenin pathway activation during early persister cell enrichment.

A) Principal Component Analysis (PCA) plot of MDA-MB-231 cell line treated with DOC or CAR for 72h. **B-C)** Volcano plots of differentially regulated (down- (left) and up- (right) regulated) genes for MDA-MB-231 cell line treated with DOC or CAR. Gene values are reported as Log2FoldChange. **D-E)** Heatmaps of normalized expression for ~12-20 representative genes (selected from DEGs predictive of deregulated processes and/or hallmarks) in MDA-MB-231 cell line treated with DOC or CAR. Processes and genes shown in panels **D-E** were based on enriched gene sets from Hallmark databases analyzed by one-tailed GSEA ranked by Normalized Enrichment Score (NES). **F)** Venn diagram of commonly downregulated (785) genes between DOC (1903) and CAR (1673) treatment (vs. UNT). **G)** Enriched Gene Ontology (GO) terms obtained by one-tailed GSEA ranked by a positive NES and based on commonly downregulated genes between DOC and CAR treatment seen in panel **F**. Red line indicates significance threshold value, (-log(NOM p-val) = 1.3). Data used to generate panels **A-G** was obtained from bulk mRNA-sequencing of MDA-MB-231 cell line treated with DOC or CAR for 72h. **H)** Immunofluorescence controls (single and unstained) of MDA-MB-231, MDA-MB-468, and PDC-BRC-101 cell lines at 72h. Scale bar, 200μm.
