# Supplementary material for: Chemotherapy-Treated Breast Cancer Cells Activate the WNT Signaling Pathway to Enter a Diapause-Like Early Persister State
Source: Cancer Res. 2025 Oct 21;86(2):310–30. doi: 10.1158/0008-5472.CAN-24-4165 (PMC12809118; doi:10.1158/0008-5472.CAN-24-4165)
Supplement: Figure S2 — SUP. Fig. 2 - Distnct chemotherapy treatments converge on robust WNT/β-catenin pathway activation during early persister cell enrichment [file can-24-4165_figure_s2_suppsf2.pdf]

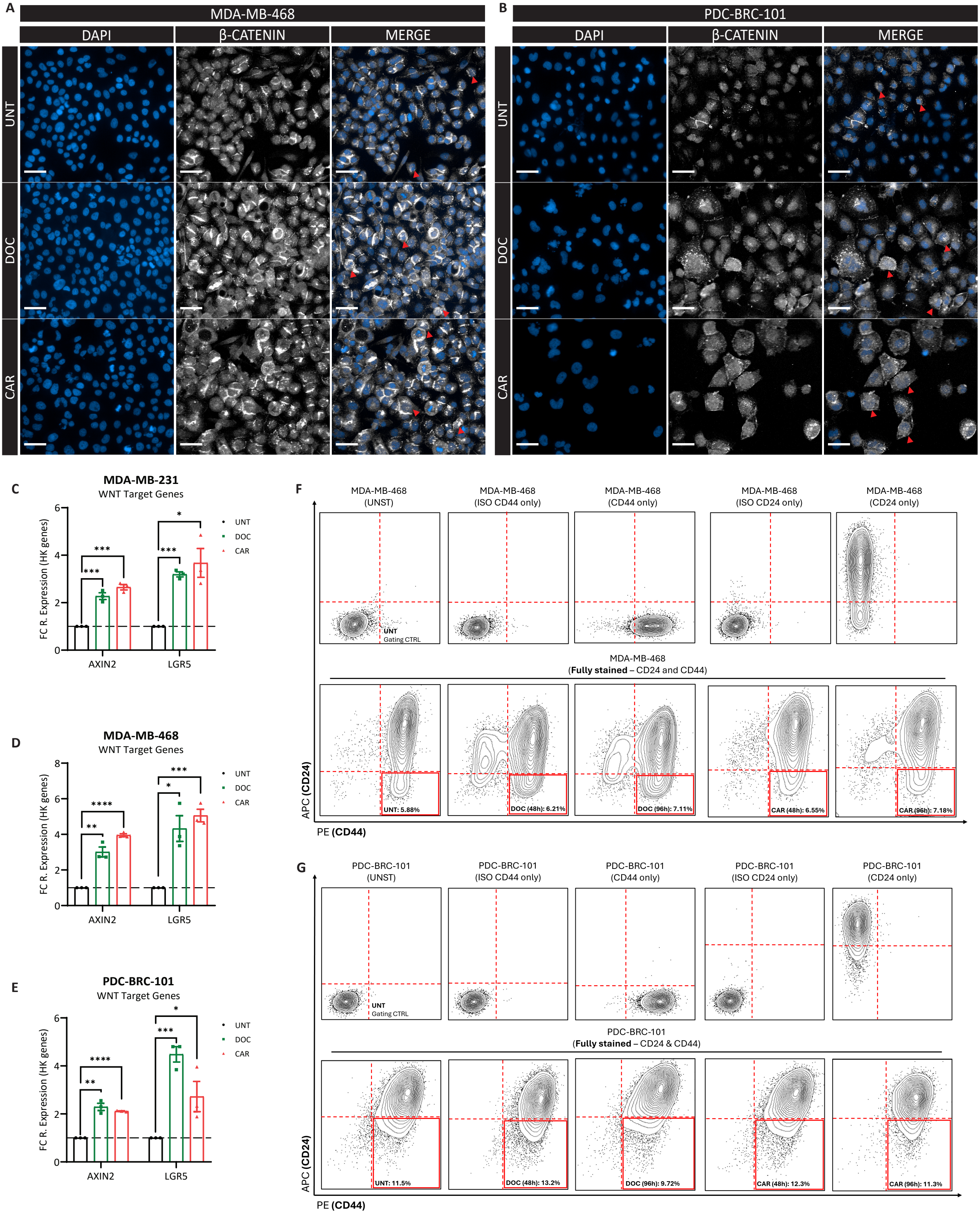

A
MDA-MB-468
DAPI
β-CATENIN
MERGE
UNT
DOC
CAR
B
PDC-BRC-101
DAPI
β-CATENIN
MERGE
UNT
DOC
CAR
C
MDA-MB-231
WNT Target Genes
FC R. Expression (HK genes)
AXIN2
LGR5
UNT
DOC
CAR
D
MDA-MB-468
WNT Target Genes
FC R. Expression (HK genes)
AXIN2
LGR5
UNT
DOC
CAR
E
PDC-BRC-101
WNT Target Genes
FC R. Expression (HK genes)
AXIN2
LGR5
UNT
DOC
CAR
F
MDA-MB-468 (UNST)
MDA-MB-468 (ISO CD44 only)
MDA-MB-468 (CD44 only)
MDA-MB-468 (ISO CD24 only)
MDA-MB-468 (CD24 only)
UNT Gating CTRL
MDA-MB-468
(Fully stained – CD24 and CD44)
UNT: 5.88%
DOC (48h): 6.21%
DOC (96h): 7.11%
CAR (48h): 6.55%
CAR (96h): 7.18%
APC (CD24)
PE (CD44)
G
PDC-BRC-101 (UNST)
PDC-BRC-101 (ISO CD44 only)
PDC-BRC-101 (CD44 only)
PDC-BRC-101 (ISO CD24 only)
PDC-BRC-101 (CD24 only)
UNT Gating CTRL
PDC-BRC-101
(Fully stained – CD24 & CD44)
UNT: 11.5%
DOC (48h): 13.2%
DOC (96h): 9.72%
CAR (48h): 12.3%
CAR (96h): 11.3%
APC (CD24)
PE (CD44)

SUP. Fig. 2: Distinct chemotherapy treatments converge on robust WNT/β-catenin pathway activation during early persister cell enrichment.

A-B) Immunofluorescence of active β-catenin in MDA-MB-468 and PDC-BRC-101 cell lines treated with DOC or CAR for 72h. Scale bar, 50µm. Images show zoomed-in regions of interest. Quantification in **Fig. 1K-M**. **C-E)** RT-qPCR of WNT target genes (*AXIN2* and *LGR5*) in TNBC cell lines treated with DOC or CAR for 72h, displayed as fold change (to UNT) of 2^{-dCt} (relative to housekeeping genes). Multiple t tests on 2^{-dCt} values, Holm-Sidak correction, n=3. Data are presented as Mean ± SEM. **F-G)** Flow cytometry analysis of %$CD24^{Low}/CD44^{High}$ cells of MDA-MB-468 and PDC-BRC-101 cell lines treated with DOC or CAR for 48h and 96h. Representative flow cytometry contour plots showing appropriate staining controls and gating strategy. Solid red lines (bottom-right quadrant) indicate population of interest; $CD24^{Low}/CD44^{High}$ cells. p values: *p < 0.05, **p < 0.01, ***p < 0.001, ****p < 0.0001, ns = not significant.
