# Supplementary material for: Chemotherapy-Treated Breast Cancer Cells Activate the WNT Signaling Pathway to Enter a Diapause-Like Early Persister State
Source: Cancer Res. 2025 Oct 21;86(2):310–30. doi: 10.1158/0008-5472.CAN-24-4165 (PMC12809118; doi:10.1158/0008-5472.CAN-24-4165)
Supplement: Figure S3 — SUP. Fig. 3 - Parental and early chemotherapy-treated WNTHigh persister cells display diapause-like cell properties. [file can-24-4165_figure_s3_suppsf3.pdf]

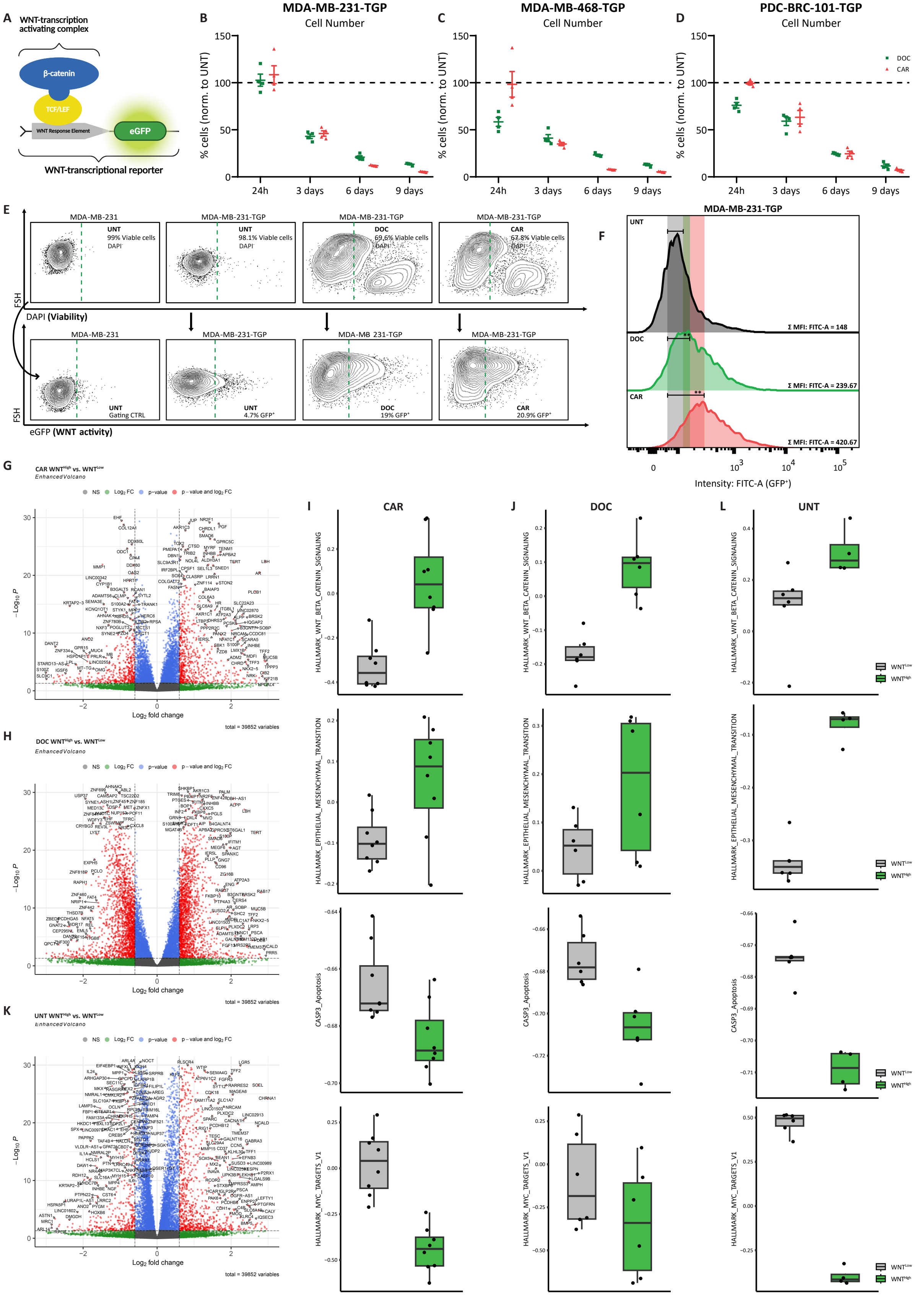
A
WNT-transcription activating complex
β-catenin
TCF/LEF
WNT Response Element
eGFP
WNT-transcriptional reporter
B
MDA-MB-231-TGP
Cell Number
C
MDA-MB-468-TGP
Cell Number
D
PDC-BRC-101-TGP
Cell Number
% cells (norm. to UNT)
24h
3 days
6 days
9 days
DOC
CAR
E
MDA-MB-231
MDA-MB-231-TGP
UNT
99% Viable cells
98.1% Viable cells
DOC
69.6% Viable cells
CAR
67.8% Viable cells
DAPI (Viability)
FSH
UNT
Gating CTRL
4.7% GFP+
19% GFP+
20.9% GFP+
eGFP (WNT activity)
F
MDA-MB-231-TGP
Σ MFI: FITC-A = 148
Σ MFI: FITC-A = 239.67
Σ MFI: FITC-A = 420.67
Intensity: FITC-A (GFP+)
G
CAR WNT^High vs. WNT^Low
EnhancedVolcano
NS
Log2 FC
p-value
p – value and log2 FC
–Log10 P
Log2 fold change
total = 39852 variables
H
DOC WNT^High vs. WNT^Low
K
UNT WNT^High vs. WNT^Low
I
CAR
J
DOC
L
UNT
HALLMARK_WNT_BETA_CATENIN_SIGNALING
HALLMARK_EPITHELIAL_MESENCHYMAL_TRANSITION
CASP3_Apoptosis
HALLMARK_MYC_TARGETS_V1
WNT^Low
WNT^High

SUP. Fig. 3: Parental and early chemotherapy-treated WNT^{High} persister cells display diapause-like cell properties.

A) Schematic representation of WNT/β-catenin transcriptional reporter (TOP-GFP/TGP). Created in BioRender. Lluis Vinas, F. (2025) https://BioRender.com/yen63o9. **B-D)** Cell counts of TNBC cell lines treated with DOC or CAR for 24h, 72h, 6days, and 9days. Dashed line represents 100% cell number (UNT culture conditions). n=4. Data are presented as Mean ± SEM. **E)** Representative flow cytometry contour plots showing FACS gating strategy, displaying %viable ($DAPI^-$) cells (left of the dashed green line – top) and % WNT^{High} (GFP^+) cells (right of the dashed green line – bottom) in MDA-MB-231-TGP cell line treated with DOC or CAR for 96h. **F)** An overlay of representative flow cytometry histograms representing the intensity of recorded GFP-expression (FITC-A channel) levels for MDA-MB-231-TGP TNBC cell line treated with DOC or CAR for 72h. Indicated numerical values denote the average median fluorescence intensity (MFI) per sample. Multiple t tests, Holm-Sidak correction, n=3, comparing MFI of DOC or CAR vs. UNT. **G-H)** Volcano plots displaying differentially regulated (down- (left) and up- (right) regulated) genes comparing sorted WNT^{High} vs. WNT^{Low} for MDA-MB-231-dTGP cell line treated with CAR or DOC. Gene values are reported as Log2FoldChange. Dot colors defined in the plot. **I-J)** Boxplots depicting the distribution of absolute scores of gene expression signatures between sorted WNT^{High} vs. WNT^{Low} cells across CAR- and DOC-treated samples. Batch effects were not accounted for in this analysis. **K)** Volcano plot displaying differentially regulated (down- (left) and up- (right) regulated) genes compar-ing sorted WNT^{High} vs. WNT^{Low} for MDA-MB-231-dTGP cell line in UNT conditions. Gene values are reported as Log2FoldChange. Dot colors defined in the plot. **L)** Box plots depicting the distribution of absolute scores of gene expression signatures between sorted WNT^{High} vs. WNT^{Low} cells in UNT conditions. Batch effects were not accounted for in this analysis. Data used to generate panels **G-L** was obtained from bulk mRNA-seq of sorted WNT^{High} and WNT^{Low} from MDA-MB-231-dTGP cell line treated with DOC or CAR for 72h. p values: *p < 0.05, **p < 0.01, ***p < 0.001, ****p < 0.0001, ns = not significant.
