# Supplementary material for: Chemotherapy-Treated Breast Cancer Cells Activate the WNT Signaling Pathway to Enter a Diapause-Like Early Persister State
Source: Cancer Res. 2025 Oct 21;86(2):310–30. doi: 10.1158/0008-5472.CAN-24-4165 (PMC12809118; doi:10.1158/0008-5472.CAN-24-4165)
Supplement: Figure S4 — SUP. Fig. 4 - WNT pathway activation triggers reduced proliferation mimicking a diapause-like state in parental TNBC cells [file can-24-4165_figure_s4_suppsf4.pdf]

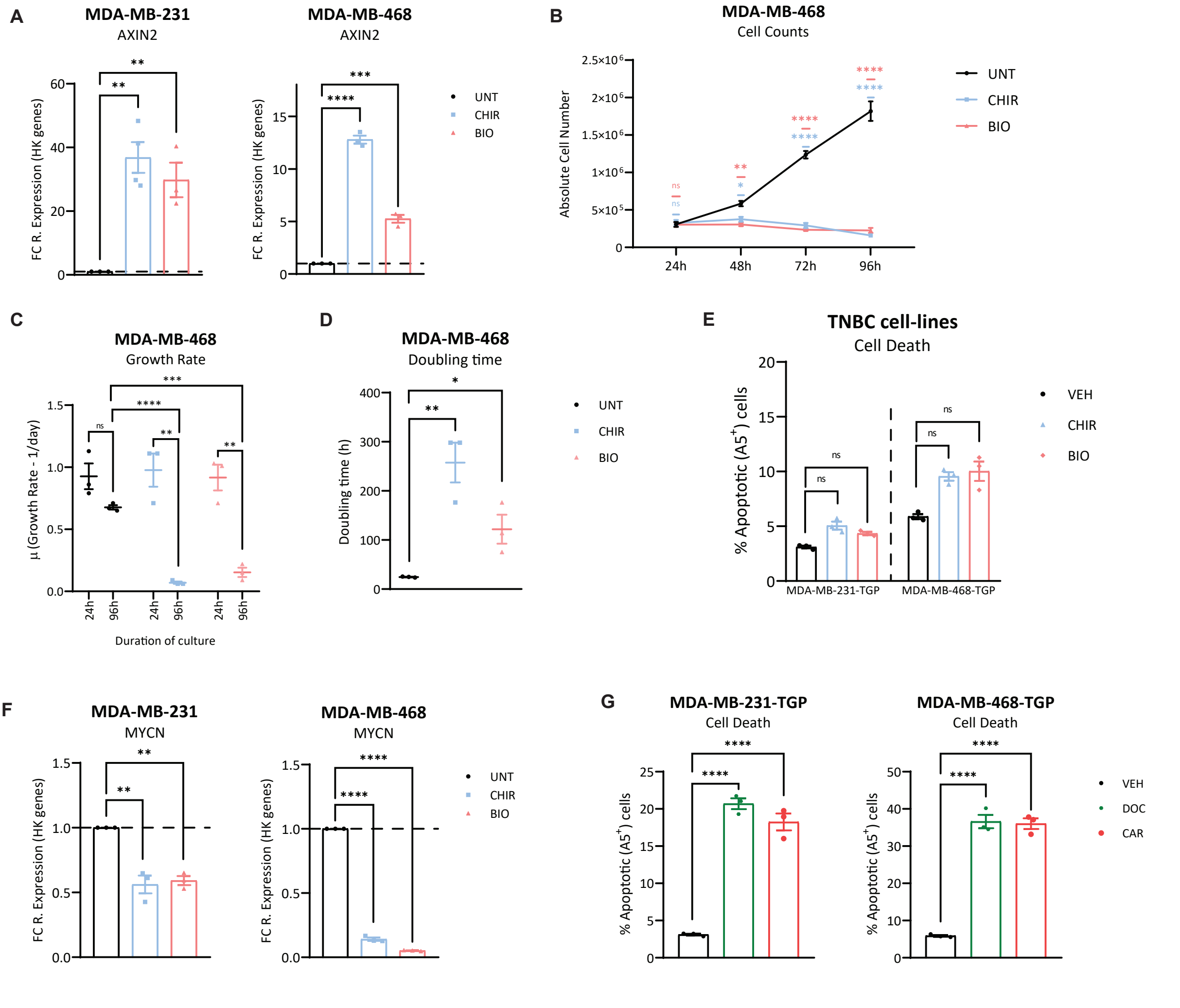

SUP. Fig. 4: WNT pathway activation triggers reduced proliferation mimicking a diapause-like state in parental TNBC cells.
A) RT-qPCR of WNT target gene (*AXIN2*) for MDA-MB-231 and MDA-MB-468 cell lines treated with CHIR or BIO for 72h, displayed as fold change (to UNT) of 2^{-dCt} (relative to housekeeping genes). Unpaired t tests on 2^{-dCt} values, n=3. **B)** Absolute cell number of MDA-MB-468 cell line treated with CHIR or BIO for a time-course of 96h. Two-way ANOVA, Tukey's correction, n=3. **C)** Growth rate of MDA-MB-468 cell line at 24h and 96h under UNT or under CHIR and BIO treatment conditions. Multiple t tests, Holm-Sidak correction, n=3. **D)** Doubling time of MDA-MB-468 cell line at 96h under UNT or under CHIR and BIO treatment conditions. Multiple t tests, Holm-Sidak correction, n=3. **E)** Flow cytometry of apoptotic (%Annexin V^+) cells in MDA-MB-231-TGP and MDA-MB-468-TGP cell lines treated with CHIR or BIO for 72h (sole treatments). One-way ANOVA, Dunnett correction, n=3. **F)** RT-qPCR of *MYCN* in MDA-MB-231 and MDA-MB-468 cell lines treated with CHIR or BIO for 72h, displayed as fold change (to UNT) of 2^{-dCt} (relative to housekeeping genes). Unpaired t tests on 2^{-dCt} values, n=3. **G)** Flow cytometry of apoptotic (%Annexin V^+) cells in MDA-MB-231-TGP (left) and MDA-MB-468-TGP (right) cell lines treated with chemotherapy (DOC or CAR) for 72h (sole treatments). One-way ANOVA, Dunnett correction, n=3. Unless specified otherwise, all data is presented as Mean ± SEM. p values: *p < 0.05, **p < 0.01, ***p < 0.001, ****p < 0.0001, ns = not significant.
