# Supplementary material for: Chemotherapy-Treated Breast Cancer Cells Activate the WNT Signaling Pathway to Enter a Diapause-Like Early Persister State
Source: Cancer Res. 2025 Oct 21;86(2):310–30. doi: 10.1158/0008-5472.CAN-24-4165 (PMC12809118; doi:10.1158/0008-5472.CAN-24-4165)
Supplement: Figure S5 — SUP. Fig. 5 - Induction of transient de novo WNT signaling transcriptional activation in response to chemotherapy in in vitro TNBC cell lines and in an in vivo TNBC xenograph model. [file can-24-4165_figure_s5_suppsf5.pdf]

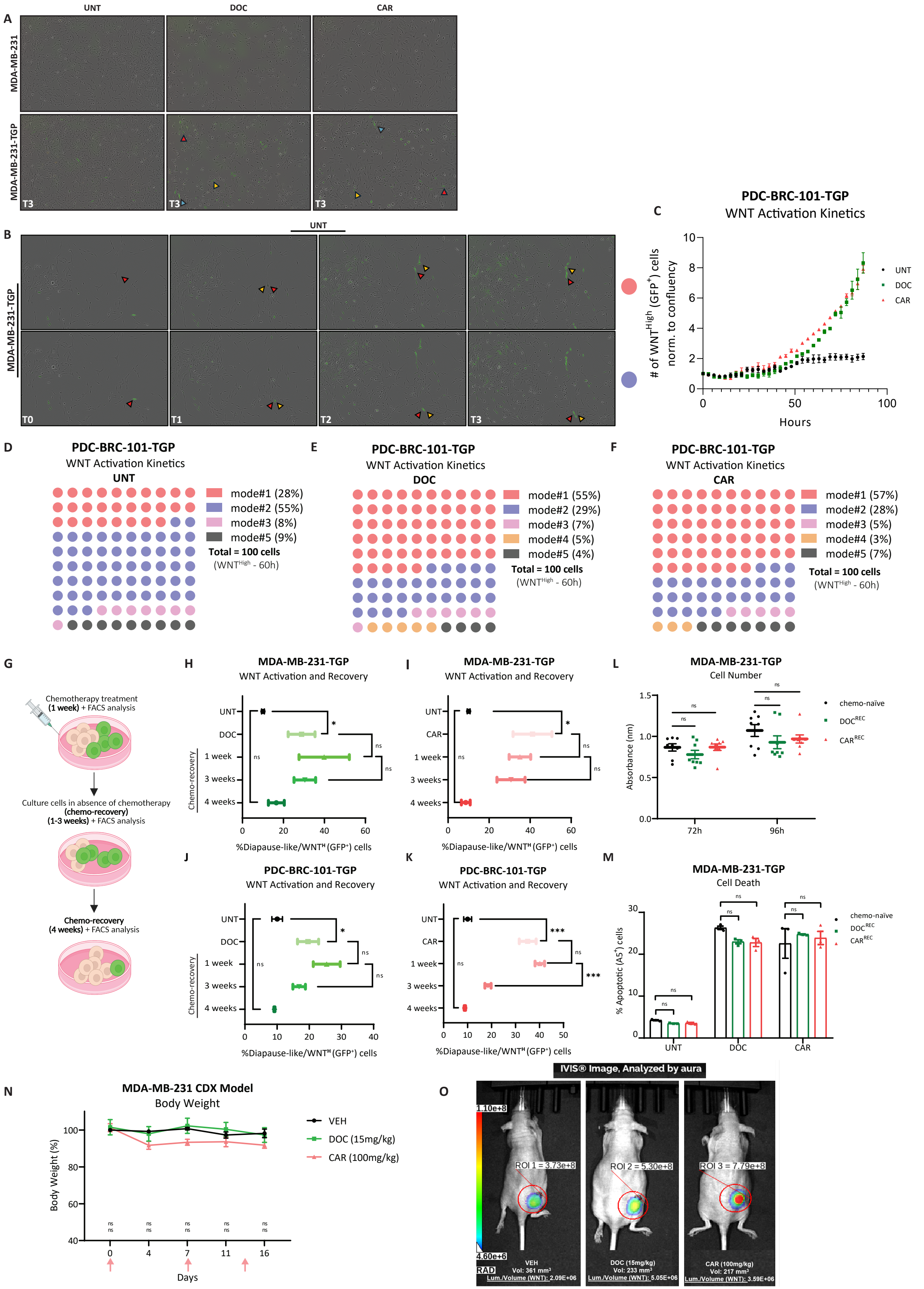

A
UNT
DOC
CAR
MDA-MB-231
MDA-MB-231-TGP
T3
B
UNT
MDA-MB-231-TGP
T0
T1
T2
T3
C
PDC-BRC-101-TGP
WNT Activation Kinetics
# of WNT^High (GFP+) cells norm. to confluency
Hours
UNT
DOC
CAR
D
PDC-BRC-101-TGP
WNT Activation Kinetics
UNT
mode#1 (28%)
mode#2 (55%)
mode#3 (8%)
mode#5 (9%)
Total = 100 cells
(WNT^High - 60h)
E
PDC-BRC-101-TGP
WNT Activation Kinetics
DOC
mode#1 (55%)
mode#2 (29%)
mode#3 (7%)
mode#4 (5%)
mode#5 (4%)
Total = 100 cells
(WNT^High - 60h)
F
PDC-BRC-101-TGP
WNT Activation Kinetics
CAR
mode#1 (57%)
mode#2 (28%)
mode#3 (5%)
mode#4 (3%)
mode#5 (7%)
Total = 100 cells
(WNT^High - 60h)
G
Chemotherapy treatment
(1 week) + FACS analysis
Culture cells in absence of chemotherapy
(chemo-recovery)
(1-3 weeks) + FACS analysis
Chemo-recovery
(4 weeks) + FACS analysis
H
MDA-MB-231-TGP
WNT Activation and Recovery
UNT
DOC
1 week
3 weeks
4 weeks
Chemo-recovery
%Diapause-like/WNT^H (GFP+) cells
I
MDA-MB-231-TGP
WNT Activation and Recovery
UNT
CAR
1 week
3 weeks
4 weeks
%Diapause-like/WNT^H (GFP+) cells
L
MDA-MB-231-TGP
Cell Number
Absorbance (nm)
72h
96h
chemo-naïve
DOC^REC
CAR^REC
J
PDC-BRC-101-TGP
WNT Activation and Recovery
%Diapause-like/WNT^H (GFP+) cells
K
PDC-BRC-101-TGP
WNT Activation and Recovery
%Diapause-like/WNT^H (GFP+) cells
M
MDA-MB-231-TGP
Cell Death
% Apoptotic (A5+) cells
UNT
DOC
CAR
chemo-naïve
DOC^REC
CAR^REC
N
MDA-MB-231 CDX Model
Body Weight
Body Weight (%)
Days
VEH
DOC (15mg/kg)
CAR (100mg/kg)
O
IVIS® Image, Analyzed by aura
1.10e+8
4.60e+6
RAD
ROI 1 = 3.73e+8
ROI 2 = 5.30e+8
ROI 3 = 7.79e+8
VEH
Vol: 361 mm3
Lum./Volume (WNT): 2.09E+06
DOC (15mg/kg)
Vol: 233 mm3
Lum./Volume (WNT): 5.05E+06
CAR (100mg/kg)
Vol: 217 mm3
Lum./Volume (WNT): 3.59E+06

SUP. Fig. 5: Induction of transient *de novo* WNT signaling transcriptional activation in response to chemotherapy in *in vitro* TNBC cell lines and in an *in vivo* TNBC xenograft model.
A) Time-lapse stills of MDA-MB-231 and MDA-MB-231-TGP cell lines under UNT, DOC, and CAR conditions. T_3 indicates 60h. Yellow, red, and blue arrows indicate WNT^{High} (GFP^+) cells. MDA-MB-231 cell line lacking the integrated WNT reporter (TGP) was used as a control for background fluorescence signal. **B)** Still frames from live imaging experiments of MDA-MB-231-TGP TNBC cell line in UNT conditions presenting mode #1 and mode #2 (color coding in scheme shown in **Fig. 4B**). T_0 indicates 0h, T_1 indicates 30h, T_2 indicates 48h, and T_3 indicates 60h. Yellow, red, and blue arrows indicate the same cell followed over the culturing period spanning different images (horizontal). **C)** Number of WNT^{High} (GFP^+) cells by live-cell imaging, normalized to the confluency, in PDC-BRC-101-TGP cell line treated with DOC or CAR. **D-F)** Quantification of WNT activation dynamics in PDC-BRC-101-TGP cell line (UNT, DOC, or CAR). n=100 cells tracked every 2h for 60h; each circle = one cell, color-coded as in **Fig. 4B**. **G)** Schematic representation of the experimental setup for chemotherapy treatment and recovery. Created in BioRender. Lluis Vinas, F. (2025) https://BioRender.com/rcrppmp. **H-K)** Flow cytometry analysis of %Diapause-like/WNT^{High} (GFP^+) within viable ($DAPI^-$) cells in MDA-MB-231-TGP and PDC-BRC-101-TGP cell lines treated with DOC or CAR for 1 week followed by 1-, 3-, or 4-week recovery in chemotherapy-free conditions. Two-tailed unpaired t tests, n=3. **L)** Metabolic activity levels reflecting cell number and proliferation rates of MDA-MB-231-TGP cell line (chemo-naïve vs. DOC^{REC} and CAR^{REC}), under UNT conditions at 72h and 96h. Two-way ANOVA, Tukey's correction, n=3. **M)** Flow cytometry of apoptotic (%Annexin V^+) cells in MDA-MB-231-TGP cell line (chemo-naïve vs. DOC^{REC} and CAR^{REC}), under UNT conditions or treated with DOC or CAR for 72h. Two-way ANOVA, Tukey's correction, n=3. **N)** % change in body weight relative to baseline (initial) weight in xenografts treated with VEH, DOC, or CAR. Two-way ANOVA, Fisher's LSD test, n=8-7 mice per treatment group. **O)** Representative IVIS bioluminescent images displaying one mouse from each treatment group (VEH – left, DOC – middle, and CAR – right) with corresponding tumor volume and calculated WNT activation signal. Unless specified otherwise, all data is presented as Mean ± SEM. p values: *$p < 0.05$, **$p < 0.01$, ***$p < 0.001$, ****$p < 0.0001$, ns = not significant.
