# Supplementary material for: Chemotherapy-Treated Breast Cancer Cells Activate the WNT Signaling Pathway to Enter a Diapause-Like Early Persister State
Source: Cancer Res. 2025 Oct 21;86(2):310–30. doi: 10.1158/0008-5472.CAN-24-4165 (PMC12809118; doi:10.1158/0008-5472.CAN-24-4165)
Supplement: Figure S6 — SUP. Fig. 6 - Chemotherapeutic treatment induces elevated transcriptional expression of WNT ligands, WNT enhancers, and WNT secretion machinery components [file can-24-4165_figure_s6_suppsf6.pdf]

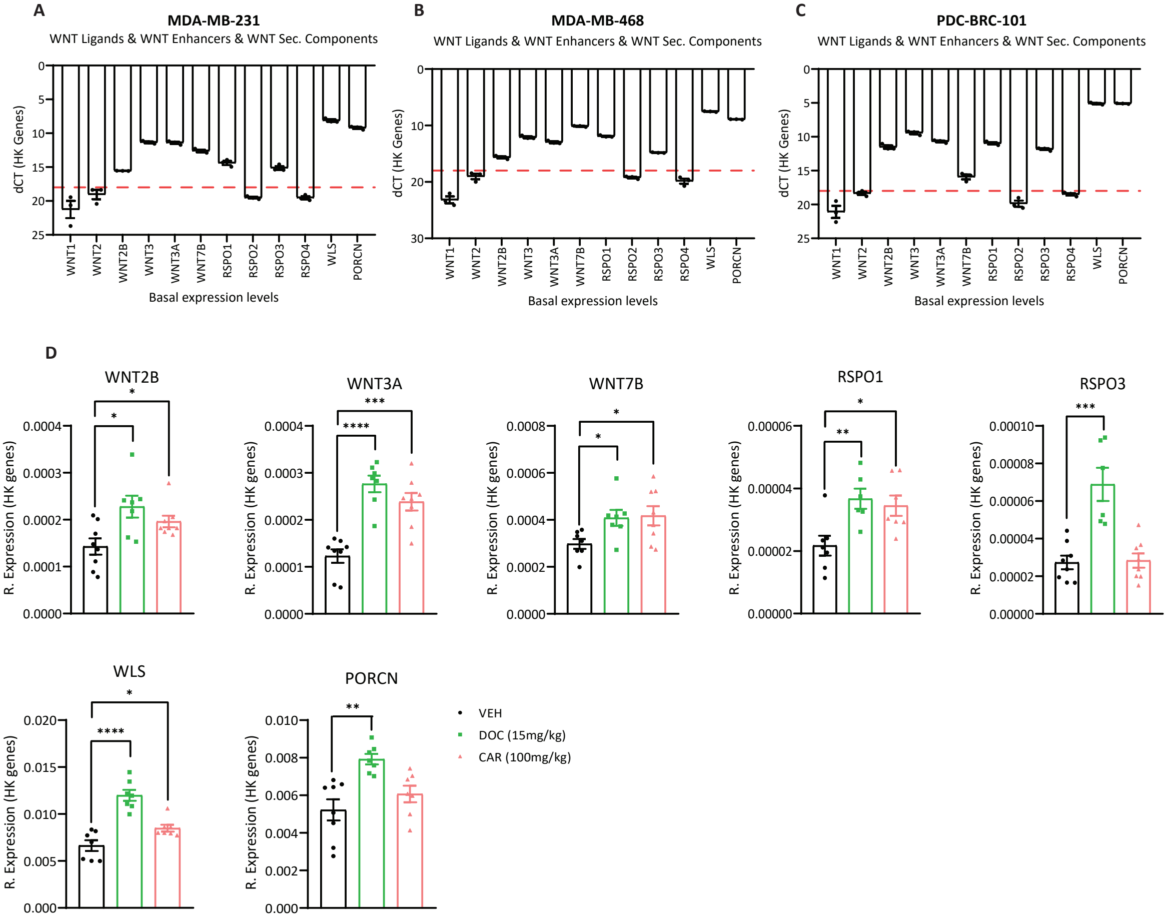

SUP. Fig. 6: Chemotherapeutic treatment induces elevated transcriptional expression of WNT ligands, WNT enhancers, and WNT secretion machinery components.

A-C) RT-qPCR of WNT ligands, WNT enhancers, and WNT secretion machinery components in TNBC cell lines under basal (UNT) culture conditions displayed as dCt values (relative to housekeeping genes), n=3. The dashed red line represents a threshold dCt value of 18. Genes that record dCt values above 18 are not considered robustly expressed and are therefore not subject to further analysis in our study. **D)** RT-qPCR of WNT activators (WNT ligands, enhancers, and secretion machinery components) in samples resected from xenograft models **Fig. 4H** treated with VEH, DOC, or CAR, displayed as 2^{-dCt} (relative to housekeeping genes). Unpaired t tests on 2^{-dCt} values, n=8-7 mice per treatment group. Unless specified otherwise, all data is presented as Mean ± SEM. p values: *p < 0.05, **p < 0.01, ***p < 0.001, ****p < 0.0001, ns = not significant.
