# Supplementary material for: Chemotherapy-Treated Breast Cancer Cells Activate the WNT Signaling Pathway to Enter a Diapause-Like Early Persister State
Source: Cancer Res. 2025 Oct 21;86(2):310–30. doi: 10.1158/0008-5472.CAN-24-4165 (PMC12809118; doi:10.1158/0008-5472.CAN-24-4165)
Supplement: Figure S7 — SUP. Fig. 7 - WNT ligand secretion-inhibition alongside chemotherapeutic treatment hinders diapause-like early persister cell enrichment in vitro and synergistically sensitizes an in vivo TNBC xenograph model [file can-24-4165_figure_s7_suppsf7.pdf]

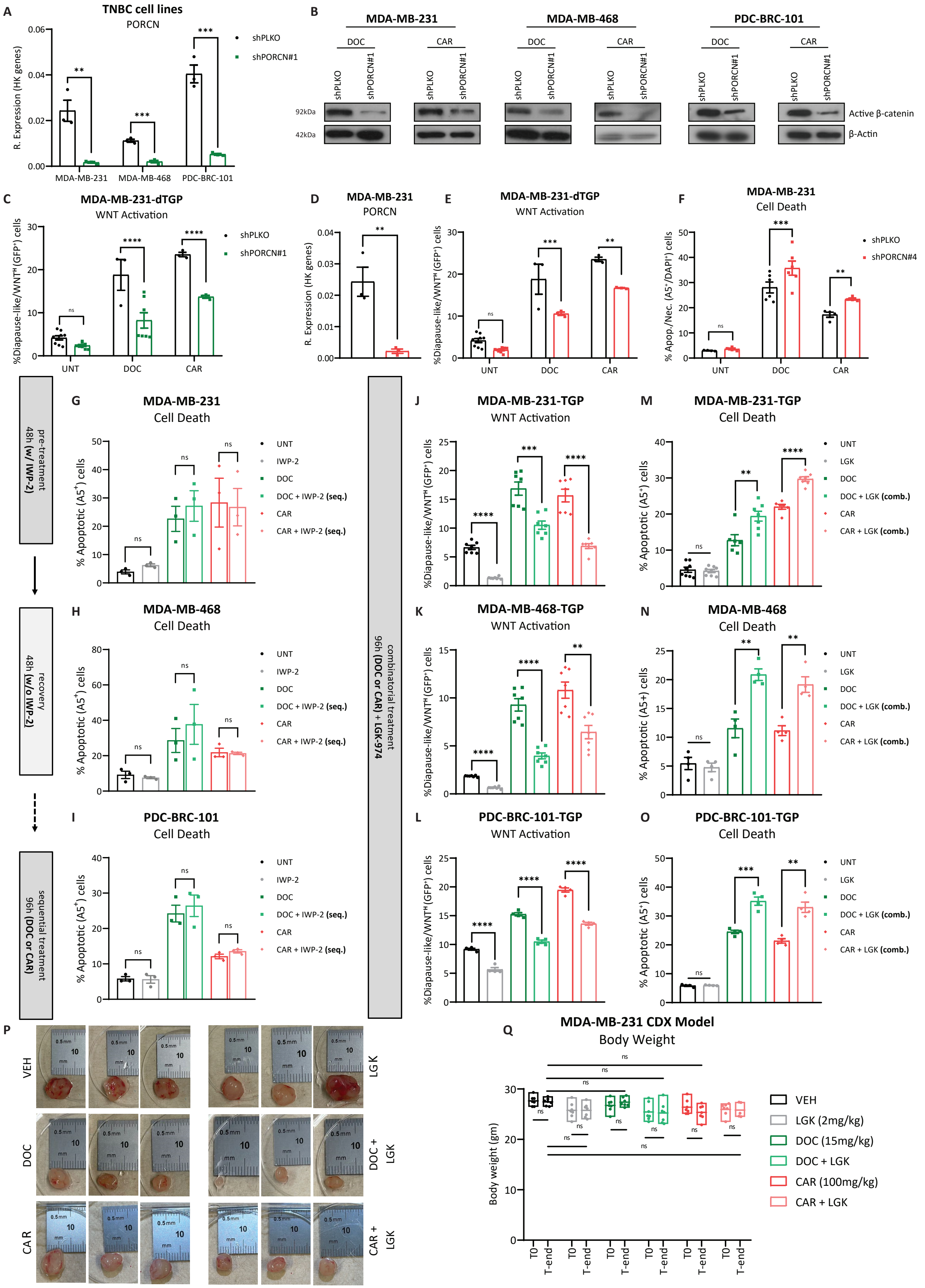

A
TNBC cell lines
PORCN
shPLKO
shPORCN#1
R. Expression (HK genes)
MDA-MB-231
MDA-MB-468
PDC-BRC-101
B
MDA-MB-231
MDA-MB-468
PDC-BRC-101
DOC
CAR
shPLKO
shPORCN#1
92kDa
42kDa
Active β-catenin
β-Actin
C
MDA-MB-231-dTGP
WNT Activation
%Diapause-like/WNT^{H}(GFP^{+}) cells
UNT
DOC
CAR
D
MDA-MB-231
PORCN
E
MDA-MB-231-dTGP
WNT Activation
F
MDA-MB-231
Cell Death
% Apop./Nec. (A5^{+}/DAPI^{+}) cells
shPORCN#4
pre-treatment
48h (w/ IWP-2)
recovery
48h (w/o IWP-2)
sequential treatment
96h (DOC or CAR)
G
MDA-MB-231
Cell Death
% Apoptotic (A5^{+}) cells
UNT
IWP-2
DOC
DOC + IWP-2 (seq.)
CAR
CAR + IWP-2 (seq.)
H
MDA-MB-468
Cell Death
I
PDC-BRC-101
Cell Death
combinatorial treatment
96h (DOC or CAR) + LGK-974
J
MDA-MB-231-TGP
WNT Activation
K
MDA-MB-468-TGP
WNT Activation
L
PDC-BRC-101-TGP
WNT Activation
M
MDA-MB-231-TGP
Cell Death
LGK
DOC + LGK (comb.)
CAR + LGK (comb.)
N
MDA-MB-468
Cell Death
% Apoptotic (A5+) cells
O
PDC-BRC-101-TGP
Cell Death
P
VEH
DOC
CAR
LGK
DOC + LGK
CAR + LGK
Q
MDA-MB-231 CDX Model
Body Weight
Body weight (gm)
T0
T-end
VEH
LGK (2mg/kg)
DOC (15mg/kg)
DOC + LGK
CAR (100mg/kg)
CAR + LGK

SUP. Fig. 7: WNT ligand secretion-inhibition alongside chemotherapeutic treatment hinders diapause-like early persister cell enrichment *in vitro* and synergistically sensitizes an *in vivo* TNBC xenograft model.

A) RT-qPCR of *PORCN* in TNBC cell lines (shPLKO vs. shPORCN#1) under basal conditions, displayed as 2^{-dCt} (relative to housekeeping genes). Multiple t tests on 2^{-dCt} values, Holm-Sidak correction, n=3. **B)** Western blots of active (non-phosphorylated) β-catenin in TNBC cell lines (shPLKO vs. shPORCN#1) treated with DOC or CAR for 96h. **C)** Flow cytometry of %Diapause-like/WNT^{High} (GFP^+) cells within viable ($DAPI^-$) cells in MDA-MB-231-dTGP cell line (shPLKO vs. shPORCN#1) treated with DOC or CAR for 96h. Multiple t tests, Holm-Sidak correction, n=3-4. **D)** RT-qPCR of *PORCN* of MDA-MB-231 cell line (shPLKO vs. shPORCN#4) under basal culture conditions, displayed as 2^{-dCt} (relative to housekeeping genes). Unpaired t tests on 2^{-dCt} values, n=3. **E)** Flow cytometry of %Diapause-like/WNT^{High} (GFP^+) cells within viable ($DAPI^-$) MDA-MB-231-dTGP cell line (shPLKO vs. shPORCN#4) treated with DOC or CAR for 96h. Multiple t tests, Holm-Sidak correction, n=3-4. **F)** Flow cytometry of apoptotic and necrotic (%Annexin V^+/$DAPI^+$) cells in MDA-MB-231 cell line (shPLKO vs. shPORCN#4) treated with DOC or CAR for 96h. Multiple t tests, Holm-Sidak correction, n=4. **G-I)** Flow cytometry of apoptotic (%Annexin V^+) cells in TNBC cell lines pretreated with IWP-2 (10µM, 48h) or treated with DOC or CAR for 96h (sole or sequential with IWP-2). Multiple t tests, Holm-Sidak correction, n=3. **J-L)** Flow cytometry of %Diapause-like/WNT^{High} (GFP^+) cells within viable ($DAPI^-$) TNBC-TGP cell lines treated with DOC or CAR for 96h (sole or in combination with LGK-974, 2µM). Multiple t tests, Holm-Sidak correction, n=4. **M-O)** Flow cytometry of apoptotic (%Annexin V^+) cells in TNBC-TGP cell lines treated with DOC or CAR for 96h (sole or in combination with LGK-974). Multiple t tests, Holm-Sidak correction, n=4. **P)** Representative images (3 per treatment group) of resected tumors from xenograft models treated with VEH, LGK, DOC, DOC+LGK, CAR, or CAR+LGK. **Q)** Body weight measurements evaluated at T0 (onset of treatment) and T-end (day of sacrifice) of xenograft models treated with VEH, LGK, DOC, DOC+LGK, CAR, or CAR+LGK. Two-way ANOVA, Tukey's correction, n: VEH = 8, LGK = 7, DOC = 7, DOC + LGK = 6, CAR = 7, and CAR + LGK = 5 animals. Unless specified otherwise, all data is presented as Mean ± SEM. p values: $^*p < 0.05$, $^{**}p < 0.01$, $^{***}p < 0.001$, $^{****}p < 0.0001$, ns = not significant.
