# Supplementary material for: Chemotherapy-Treated Breast Cancer Cells Activate the WNT Signaling Pathway to Enter a Diapause-Like Early Persister State
Source: Cancer Res. 2025 Oct 21;86(2):310–30. doi: 10.1158/0008-5472.CAN-24-4165 (PMC12809118; doi:10.1158/0008-5472.CAN-24-4165)
Supplement: Figure S8 — SUP. Fig. 8 - Preclinical PDO models recapitulate chemotherapy-mediated WNT activation and sensitization to synergistic WNT ligand secretion-inhibition [file can-24-4165_figure_s8_suppsf8.pdf]

A

Sample ID	Tumor	Treatment Status	Treatment	Response	EUSOMA
R1-IDC113	Primary Breast	Pretreated	ddAC	Progression	EUSOMA 3
R2-IDC159A	Primary Breast	Pretreated	AC + paclitaxel + carboplatin + fulvestrant + radiotherapy	Progression	EUSOMA 3

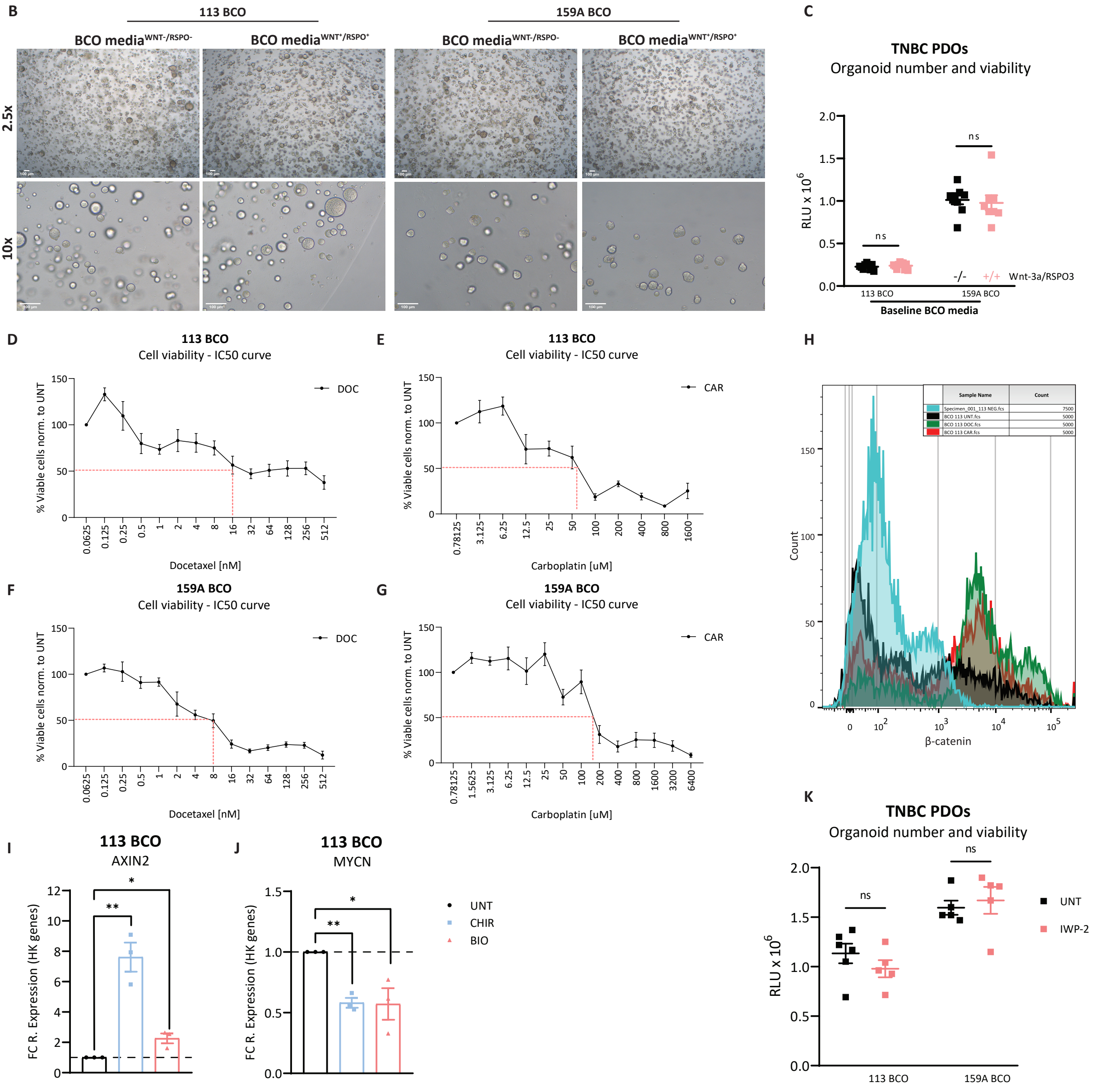

SUP. Fig. 8: Preclinical PDO models recapitulate chemotherapy-mediated WNT activation and sensitization to synergistic WNT ligand secretion-inhibition.

A) Overview of supplementary information for the TNBC PDO models used in the study. **B)** Phase-contrast images of TNBC-PDO models, 113 BCO (left) and 159A BCO (right) cultured in media not-supplemented with WNT-RSPO (BCO media$^{WNT-/RSPO-}$) and media supplemented with WNT-RSPO (BCO media$^{WNT+/RSPO+}$), shown at 2.5x (top) and 10x (bottom) magnification. **C)** Cell number and viability of 113 (left) and 159A (right) BCO models cultured in media not-supplemented with WNT-RSPO (BCO media$^{-/-}$) and media supplemented with WNT-RSPO (BCO media$^{+/+}$). Unpaired t tests, n=3. **D-G)** Drug dose-response curves of 113 and 159A BCO models treated with increasing concentrations DOC or CAR for 96h. Dashed line represents 50% viability (normalized to UNT/basal culture conditions). **H)** Representative histograms obtained from flow cytometry β-catenin staining displaying counts and intensity of β-catenin signal in 113 BCO model treated with DOC or CAR for 96h. Light blue signal displays background signal obtained from an unstained (NEG) sample. **I-J)** RT-qPCR of WNT target gene (*AXIN2*) and *MYCN* in 113 BCO model treated with CHIR or BIO for 72h, displayed as fold change (to UNT) of 2^{-dCt} (relative to housekeeping genes). Unpaired t tests on 2^{-dCt} values, n=3. **K)** Cell number and viability of 113 (left) and 159A (right) BCO models cultured in basal conditions and treated with IWP-2 (50µM) for 96h. Unpaired t tests, n=3. Unless specified otherwise, all data is presented as Mean ± SEM. p values: *p < 0.05, **p < 0.01, ***p < 0.001, ****p < 0.0001, ns = not significant.
